# Phylogenetics-based identification and characterization of a superior 2,3-butanediol dehydrogenase for *Zymomonas mobilis* expression

**DOI:** 10.1186/s13068-020-01820-x

**Published:** 2020-11-10

**Authors:** Venkataramanan Subramanian, Vladimir V. Lunin, Samuel J. Farmer, Markus Alahuhta, Kyle T. Moore, Angela Ho, Yogesh B. Chaudhari, Min Zhang, Michael E. Himmel, Stephen R. Decker

**Affiliations:** 1 Biosciences Center, National Renewable Energy Laboratory, 15013 Denver West Parkway, Golden, CO 80401 USA; 2 Biodiversity and Ecosystem Research, Institute of Advanced Study in Science and Technology (IASST), Guwahati, Assam India

**Keywords:** Butanediol dehydrogenase, *Serratia marcescens*, Acetoin, 2,3-Butanediol, Crystallography, Phylogenetics

## Abstract

**Background:**

*Zymomonas mobilis* has recently been shown to be capable of producing the valuable platform biochemical, 2,3-butanediol (2,3-BDO). Despite this capability, the production of high titers of 2,3-BDO is restricted by several physiological parameters. One such bottleneck involves the conversion of acetoin to 2,3-BDO, a step catalyzed by 2,3-butanediol dehydrogenase (Bdh). Several Bdh enzymes have been successfully expressed in *Z. mobilis,* although a highly active enzyme is yet to be identified for expression in this host. Here, we report the application of a phylogenetic approach to identify and characterize a superior Bdh, followed by validation of its structural attributes using a mutagenesis approach.

**Results:**

Of the 11 distinct *bdh* genes that were expressed in *Z. mobilis,* crude extracts expressing *Serratia marcescens* Bdh (*Sm*Bdh) were found to have the highest activity (8.89 µmol/min/mg), when compared to other Bdh enzymes (0.34–2.87 µmol/min/mg). The *Sm*Bdh crystal structure was determined through crystallization with cofactor (NAD^+^) and substrate (acetoin) molecules bound in the active site. Active *Sm*Bdh was shown to be a tetramer with the active site populated by a Gln247 residue contributed by the diagonally opposite subunit. *Sm*Bdh showed a more extensive supporting hydrogen-bond network in comparison to the other well-studied Bdh enzymes, which enables improved substrate positioning and substrate specificity. This protein also contains a short α6 helix, which provides more efficient entry and exit of molecules from the active site, thereby contributing to enhanced substrate turnover. Extending the α6 helix to mimic the lower activity *Enterobacter cloacae* (*Ec*Bdh) enzyme resulted in reduction of *Sm*Bdh function to nearly 3% of the total activity. In great contrast, reduction of the corresponding α6 helix of the *Ec*Bdh to mimic the *Sm*Bdh structure resulted in ~ 70% increase in its activity.

**Conclusions:**

This study has demonstrated that *Sm*Bdh is superior to other Bdhs for expression in *Z. mobilis* for 2,3-BDO production. *Sm*Bdh possesses unique structural features that confer biochemical advantage to this protein. While coordinated active site formation is a unique structural characteristic of this tetrameric complex, the smaller α6 helix and extended hydrogen network contribute towards improved activity and substrate promiscuity of the enzyme.

## Background

Petroleum alternatives are critical to maintaining a sustainable economy while satisfying the ever-growing global energy demands. Diols, potentially renewable compounds containing two hydroxyl groups, have wide-ranging applications in chemicals and fuels. 2,3-butanediol (2,3-BDO) is exemplar of industrial diols having been used in liquid fuels, cosmetics and drugs, paints, food additives, and synthetic rubber [1, 2].

With two chiral centers, three isomers of 2,3-BDO are possible. The *levo* (2*R*, 3*R*) and *dextro* (2*S*, 3*S*) isomers are optically active, whereas the *meso* (2*R*, 3*S*) isomer is not [3, 4]. Biological synthesis of 2,3-BDO occurs via three enzymatic conversion steps [5, 6]. The first step involves condensation of two pyruvate molecules by the acetolactate synthase (Als) in a single decarboxylation reaction to produce α-acetolactate. A second decarboxylation step catalyzed by the enzyme acetolactate-decarboxylase produces acetoin. In the presence of NADH, acetoin is reduced to 2,3-BDO by the enzyme butanediol dehydrogenase (Bdh). Under aerobic conditions, the acetoin spontaneously converts to diacetyl, which can be reconverted to acetoin by diacetyl reductase and subsequently reduced to 2,3-BDO. Depending on oxygenation conditions, (3R)-acetoin or (3S)-acetoin is preferably produced by microorganisms. (3R)-acetoin is the product of anaerobic fermentation from acetolactate. However, under aerobic conditions, (3S)-acetoin is produced from diacetyl [7]. Depending on the type of acetoin used, three different stereoisomers of 2,3-BDO, (2R, 3R)-BDO, (2R-3S)-BDO, and (2S, 3S)-BDO are produced [8].

Several bacteria, including *Klebsiella* sps. *Enterobacter* sps., *Pseudomonas* sps., *Serratia* sp., and *Bacillus* sps. are known to naturally produce 2,3-BDO. Interestingly, the highest 2,3-BDO concentrations are produced by risk group 2 organisms and are thus not desirable for large-scale production [4, 9]. Organisms, such as *Escherichia coli*, *Lactobacillus lactis*, *Synechococcus elongatus*, and even yeasts such as *Saccharomyces cerevisiae* have been engineered to produce 2,3-BDO. The 2,3-BDO titers reported are 73.8, 51, 2.38, and 154.3 g/L, respectively, for these organisms [10–13].

*Zymomonas mobilis* is known primarily for its ethanologenic properties. In comparison to yeasts, which use the Entner–Meyerhof–Parnas pathway for glycolysis, *Z. mobilis* uses the Entner–Doudoroff (ED) pathway. The ED pathway is found in facultative anaerobes and aerobic microorganisms, leading to higher ethanol yields [14]. *Z. mobilis* has other advantages, such as high alcohol and pH tolerance, high rate of sugar uptake, low biomass production, and reduced aeration requirement thereby reducing the production costs [15]. Interestingly, *Z. mobilis* is capable of using both deacetylated-disc refined (DDR) and deacetylated-mechanically refined (DMR) sugar streams for ethanol production [16]. Recently, it was also demonstrated by our laboratory that the ethanol flux could be diverted to 2,3-BDO using pure or mixed sugars as substrates. By introducing the three 2,3-BDO pathway genes followed by promoter replacements and fermentation condition optimizations, the 2,3-BDO titers of > 13 g/L was achieved [15].

One of the bottlenecks for production of 2,3-BDO in *Z. mobilis* is the competition between NADH oxidase (Ndh) and Bdh to oxidize NADH to NAD^+^ under oxic conditions. This is in addition to the NADH demand for glycerol and ethanol production by this organism. Depending on the activity of the Bdh enzyme, the conversion of NADH could be shifted towards 2,3-BDO production instead of the respiratory chain. The conversion of acetoin to 2,3-BDO by Bdh is a reversible reaction governed by the pH [17, 18].

$$\mathrm{Acetoin}+NADH+{H}^{+}\stackrel{ }{\leftrightarrow } \text{2,3-butanediol}+{NAD}^{+}$$

Bdh enzymes can be classified into R-acting or S-acting depending on the chirality of the chiral center introduced by the enzyme at the acetoin C2 atom. Whereas the preference for (3R)-acetoin or (3S)-acetoin is imprinted in the geometry of the substrate-binding pocket, R-acting and S-acting Bdh enzymes belong to different protein families and possess different architectures. Recently, the first structure of an R-acting Bdh from *Bacillus subtilis* was deposited to the Protein Data Bank (PDB, www.rcsb.org, [19]) with the PDB ID 6IE0, while there are numerous structures available for other members of the medium-chain dehydrogenase/reductase (MDR) superfamily [20–23]. Examples of R-acting Bdh enzymes are those from *S. cerevisiae*, *Paenibacillus polymyxa*, *B. subtilis*, *Pseudomonas putida,* and *Bacillus licheniformis* [24–27].

S-acting Bdh enzymes belong to the short-chain dehydrogenase/reductase (SDR) superfamily of proteins [28]. The substrates for this superfamily of enzymes vary greatly in size and include glucose, alcohols, and steroids [29]. These enzymes are well studied and several S-acting Bdhs are characterized structurally, such as those of (3R)-acetoin-dependent S-acting Bdh from *Klebsiella pneumoniae* (PDB ID 1GEG, deposited in 2001) and (3S)-acetoin-dependent S-acting Bdh from *Corynebacterium glutamicum* (PDB ID 3A28, deposited in 2010) reported by Otagiri and colleagues [30, 31].

In this work, we have focused on identifying the most active Bdh enzyme that can function in *Z. mobilis* using a phylogenetics approach to enable efficient conversion of acetoin to 2,3-BDO. Furthermore, we have obtained the crystal structure of the *Serratia marcescens* Bdh (*Sm*Bdh) by expressing it in this industrially relevant host and provided comparative analysis against similar Bdh enzymes. We have also carried out structurally guided changes to *Sm*Bdh to explain its superiority over lower activity Bdh enzymes from the same family of proteins, followed by biochemical confirmation of the activity of the designed mutants.

## Results and discussion

Production of 2,3-BDO is dependent on several factors, some of which include the types of heterologous 2,3-BDO pathway genes used, the levels of oxygen present, the presence of competitive pathways, such as ethanol and glycerol, as well as competing enzymes for NADH conversion. We have concentrated on only one of these parameters, namely the activity of the NADH-consuming enzyme, Bdh, that can compete with Ndh in the presence of oxygen. This biochemical step is considered as one of the bottlenecks in 2,3-BDO production by this organism [15]. Although *Z. mobilis* has been shown to express several commonly known Bdh enzymes [15], there has been no comprehensive study undertaken to express functional Bdhs from diverse species that could contribute towards improving 2,3-BDO production in this organism. We therefore decided to take a phylogenetic approach to this problem by screening Bdh enzymes across different bacterial kingdoms to identify the best active enzyme that could be engineered in this organism.

### Identification of butanediol dehydrogenase sequences for expression in *Z. mobilis*

**Fig. 1:**
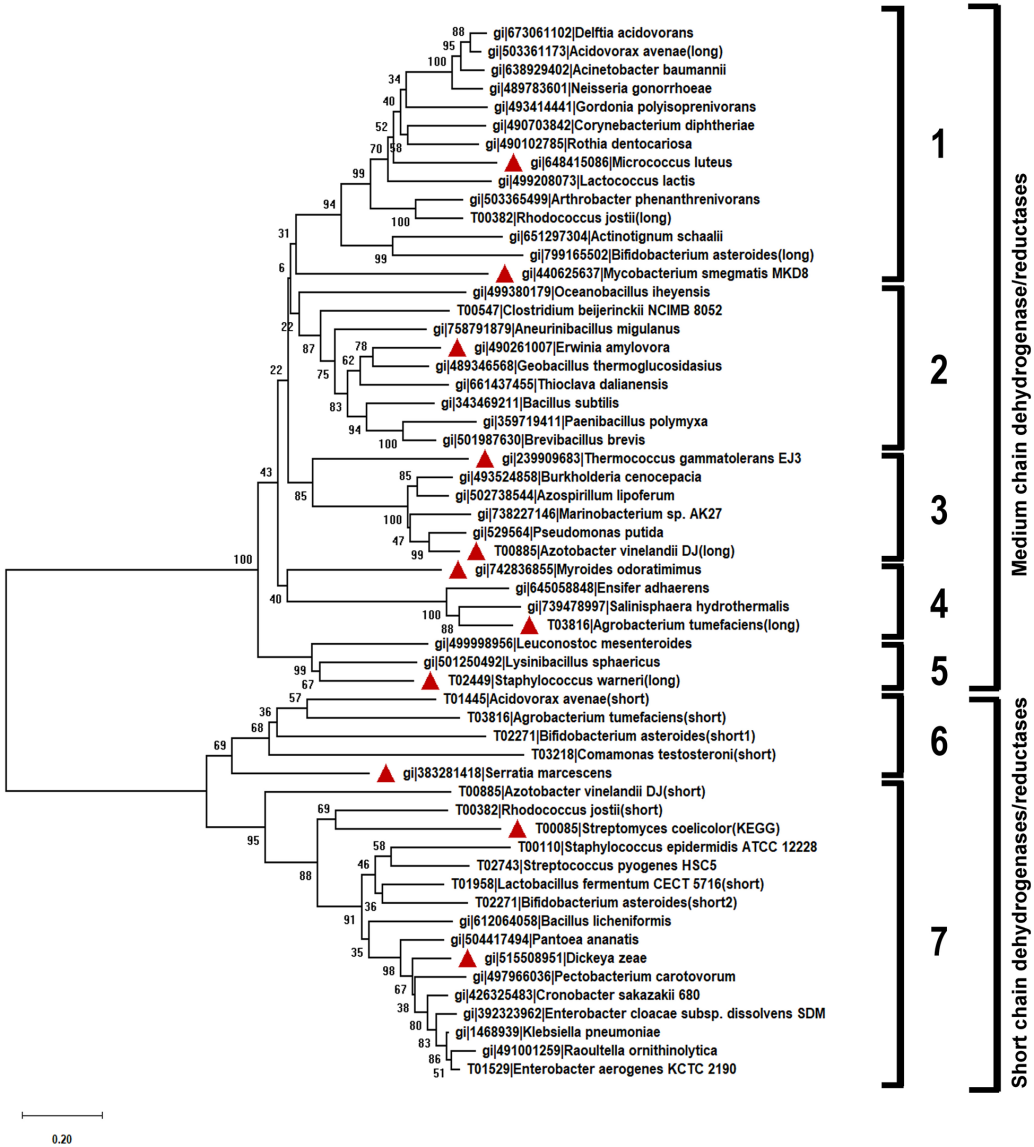
Phylogenetic tree analysis of butanediol dehydrogenases. The neighbor joining tree was generated using 57 full-length Bdh sequences. The optimal tree with the sum of branch length = 16.08413861 is shown. Boot strap values are shown next to the branches. The tree has been divided into 2 clusters, the medium-chain dehydrogenases/reductases (MDR) and short chain dehydrogenases/reductases (SDR). The MDR and SDR clusters are further subdivided into 5 and 2 sub-clusters, respectively. The sub-clusters are represented by numbers 1 through 7. Red triangles represent the sequences selected for gene synthesis

A total of 57 protein sequences were included in the list of Bdh for phylogenetic analysis. This list included two sequences each from *Azotobacter vinelandii*, *Acidovorax avenae*, *Rhodococcus jostii*, and *Agrobacterium tumefaciens*. These sequences belonged to two different classes of Bdh proteins based on their protein sequence lengths. *Bifidobacterium asteroides* contained three Bdh sequences, two belonging to the short length class and one belonging to the long class. We also included *B. subtilis, B. licheniformis, K. pneumoniae, E. cloacae*, and *S. marcescens*, studies for which expression has been carried out in *E. coli* [13]. Clearly, two distinct clusters were observed on the phylogenetic tree, one cluster (21 sequences) primarily contained the “short length” Bdhs, while the other (36 sequences) contained the “long” Bdhs (Fig. 1). The “short length” Bdh cluster comprised S-acting enzymes (presumably belonging to the SDR superfamily), such as *K. pneumoniae*, *E. cloacae* and *S. marcescens* [18, 32, 33]. The “long” Bdh cluster comprised R-acting enzymes (presumably belonging to the MDR superfamily), such as *B. subtilis* and *C. beijerinckii* [33]. Each of these two major clusters were further subdivided into smaller sub-clusters. Based on their clustering pattern, we subdivided the “short length” clusters into two sub-clusters, while subdividing the “long” class of Bdhs into five sub-clusters. Eight protein sequences were selected from the “long” and three sequences were selected from the “short-length” clusters, such that at least one representative protein sequence was selected from each sub-cluster of the phylogenetic tree (Fig. 1).

### Screening of butanediol dehydrogenases for protein expression and activity in *Z. mobilis*

The *Z. mobilis* strain used in this study was 9C, which was a modified version of strain 8b that was originally developed for improved ethanol production [15, 34]. The 9C strain lacked the chloramphenicol and tetracycline markers that the 8b strain contained. Neither of these strains were engineered for BDH production. Moreover, native *Z. mobilis* does not contain the BDH pathway to enable 2,3-BDO production. Thus, our study was not designed to carry out expression of the entire 2,3-BDO pathway, instead it was only intended to study expression and activity of the expressed BDH enzyme in this organism. Transformants obtained from the spectinomycin selection plates were confirmed for the presence of the individual *bdh* genes by PCR analysis. Eight individual transformants were subjected to colony PCR, results of which are shown in Additional file 1. Based on PCR analysis, five independent transformants were selected for protein expression analysis. Because these Bdh proteins were expressed without an epitope tag, we decided to use activity assays as a screen to detect protein expression in the different *Z. mobilis* transformants. The results of the activity assay are shown in Table 1. We observed that most of the *bdh* transformants showed an activity of ≤ 2 µmol/min/mg using acetoin and NADH as the substrate and cofactor, respectively. This included 9 of the 11 tested *bdh* genes (Additional file 2). We used three *Z. mobilis* strains expressing the *E. cloacae, B. subtilis*, and *Lactococcus lactis bdh* genes under the same promoter as controls for which expression and/or 2,3-BDO production has been tested in-house (unpublished data). Two of the Bdh enzymes, namely those from *M. luteus* and *S. marcescens*, showed Bdh activity of ~ 3.0 and 9.0 µmol/min/mg, respectively, which was 2- to 4-fold higher than the control strains. Based on the activity analysis, we selected *S. marcescens* Bdh (*Sm*Bdh) as the candidate enzyme for structural characterization. We also carried out SDS-PAGE analysis to determine expression of individual Bdh protein produced by these transformants. Interestingly, only some of the *bdh* gene transformants showed unique bands at the expected molecular weight based on Coomassie staining (Additional file 3), suggesting different expression levels of the heterologous Bdh proteins. It is likely that some of these heterologous enzymes were not expressed to detectable levels, which may have been a factor in their poor activity levels. Nevertheless, for the purpose of screening, we used their crude extract activities as the standard to determine the best-expressing and active enzyme.

**Table 1:** Enzyme activity of 2,3-butanediol dehydrogenase enzymes expressed in *Z. mobilis* strain 9C

Sample #	Origin of BDH enzyme	Activity of the crude extracts (µmol/min/mg)	Average activity of the crude extracts (µmol/min/mg)	Sample #	Origin of BDH enzyme	Activity of the crude extracts (µmol/min/mg)	Average activity of the crude extracts (µmol/min/mg)
1	Negative control	ND	ND	30	Sw1	1.13 ± 0.18	1.15 ± 0.38
				31	Sw2	1.42 ± 0.27	
2	Ec	1.79 ± 0.53	1.79 ± 0.53	32	Sw3	0.68 ± 0.13	
3	Bs	0.34 ± 0.06	0.34 ± 0.06	33	Sw4	1.71 ± 0.17	
4	Ll	1.73 ± 0.16	1.73 ± 0.16	34	Sw5	0.81 ± 0.21	
5	Dd1	1.03 ± 0.18	1.92 ± 0.51	35	Ea1	1.22 ± 0.11	1.75 ± 0.29
6	Dd2	2.47 ± 0.08		36	Ea2	2.08 ± 0.58	
7	Dd3	1.85 ± 0.39		37	Ea3	1.75 ± 0.10	
8	Dd4	1.87 ± 0.29		38	Ea4	1.79 ± 0.48	
9	Dd5	2.41 ± 0.27		39	Ea5	1.94 ± 0.13	
10	Sc1	1.71 ± 0.52	1.27 ± 0.29	40	Ml1	2.41 ± 0.89	2.87 ± 0.75
11	Sc2	1.26 ± 0.48		41	Ml2	3.09 ± 0.51	
12	Sc3	1.13 ± 0.03		42	Ml3	2.49 ± 0.27	
13	Sc4	1.42 ± 0.37		43	Ml4	2.14 ± 0.08	
14	Sc5	0.84 ± 0.05		44	Ml5	4.26 ± 0.56	
15	Sm1	6.48 ± 0.97	8.89 ± 1.43	45	Mo1	1.40 ± 0.26	1.44 ± 0.29
16	Sm2	10.95 ± 1.22		46	Mo2	1.96 ± 0.18	
17	Sm3	9.27 ± 1.21		47	Mo3	1.48 ± 0.24	
18	Sm4	9.10 ± 1.40		48	Mo4	1.07 ± 0.47	
19	Sm5	8.67 ± 0.92		49	Mo5	1.30 ± 0.38	
20	At1	1.18 ± 0.37	1.15 ± 0.59	50	Ms1	1.34 ± 0.38	1.27 ± 0.24
21	At2	1.18 ± 0.27		51	Ms2	1.22 ± 0.29	
22	At3	0.52 ± 0.11		52	Ms3	1.63 ± 0.29	
23	At4	2.22 ± 0.15		53	Ms4	0.87 ± 0.33	
24	At5	0.68 ± 0.29		54	Ms5	1.30 ± 0.20	
25	Av1	1.24 ± 0.12	1.30 ± 0.35	55	Tg1	1.09 ± 0.29	1.13 ± 0.39
26	Av2	1.92 ± 0.11		56	Tg2	0.71 ± 0.19	
27	Av3	1.40 ± 0.45		57	Tg3	1.87 ± 0.47	
28	Av4	0.91 ± 0.67		58	Tg4	0.90 ± 0.30	
29	Av5	1.05 ± 0.38		59	Tg5	1.12 ± 0.06	

Bdh activity assays were performed on whole cell extracts obtained from transformant cultures (µmol/min per mg crude extract). Each row represents activity assays performed in technical triplicates for every transformant. Five independent transformants were tested for each gene. Therefore, average specific activity has been calculated from 3 × 5 = 15 data points

Ec, *E. cloacae*; Bs, *Bacillus subtilis*; Ll, *Lactococcus lactis*; Dd, *Dickeya dadantii*; Sc, *Streptomyces coelicolor*; Sm, *Serratia marcescens*; At, *Agrobacterium tumefaciens*; Av, *Azotobacter vinelandii*; Sw, *Staphylococcus warneri*; Ea, *Erwinia amylovora*; Ml, *Micrococcus luteus*; Mo, *Myroides odoratimimus*; Ms, *Mycobacterium smegmatis*; Tg, *Thermococcus gammatolerans*

### Crystal structure of *Sm*Bdh along with its bound cofactor and substrate molecules

**Fig. 2:**
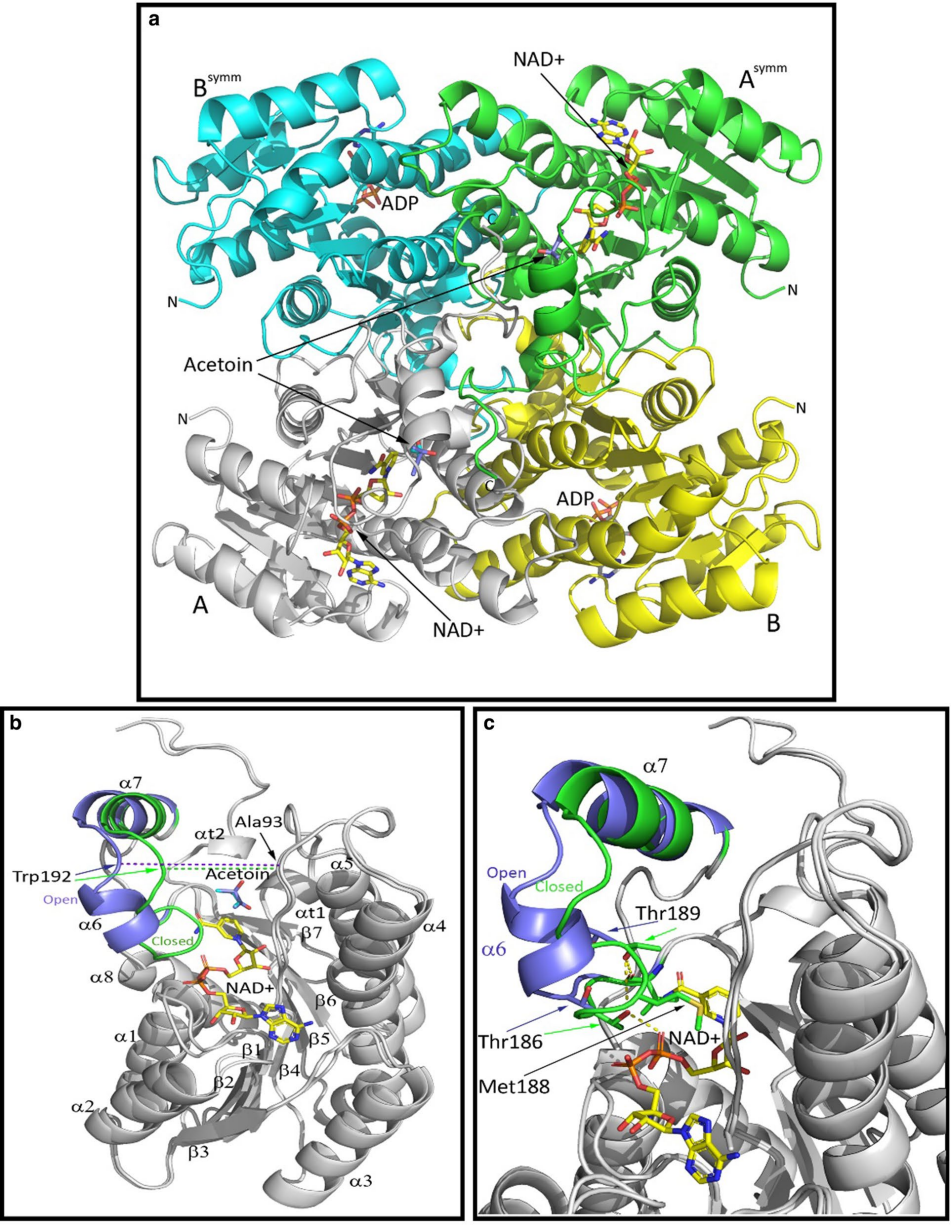
*Sm*Bdh functional complex and key structural features. **a** *Sm*Bdh tetramer. Protein chains A, B, A^symm^ and B^symm^ are shown in cartoon representation and colored grey, yellow, green and cyan, respectively. NAD^ +^ , ADP and acetoin molecules are shown in sticks representation. N- and C-termini of the protein chains are marked. **b** ‘Open’ and ‘closed’ conformations of *Sm*Bdh superimposed. Protein chains are shown in cartoon representation and colored grey except for the loop 180–203 containing helices α6 and α7. ‘Open’ conformation of the loop 180–203 is colored purple and ‘closed’ conformation is colored green. Secondary structure elements are labeled. NAD^ +^ and acetoin molecules bound in ‘closed’ conformation are shown in sticks representation. Positions of Cα atoms of Ala93 and Trp192 are shown by arrows. **c** Zoom-in view of NAD + interaction with the ‘closed’ conformation of *Sm*Bdh. Side chains of Thr186, Met188, and Thr189 are shown in sticks representation. H-bonds formed upon NAD ^+^ binding are shown as yellow dashed lines

**Fig. 3:**
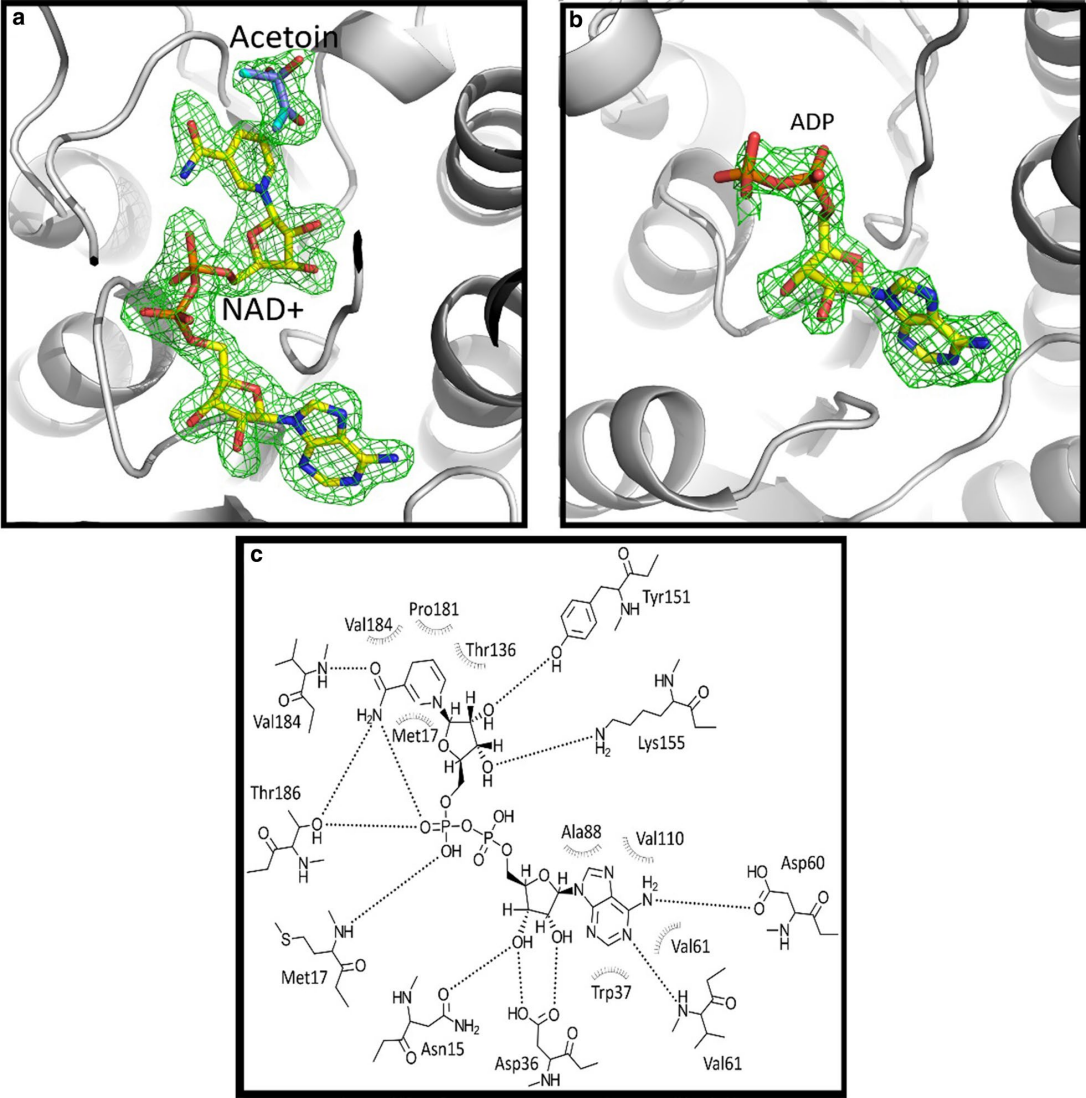
Substrate and cofactor interactions within *Sm*Bdh molecule. **a** Omit Fo-Fc electron density map (calculated after NAD ^+^ and acetoin molecules removed from the model) shown at 3σ level for the NAD^ +^ and acetoin molecules found in the ‘closed’ conformation of the WT *Sm*Bdh. **b** Omit Fo-Fc electron density map (calculated after ADP molecule was removed from the model) shown at 3σ level for the ADP molecule found in the ‘closed’ conformation of the WT *Sm*Bdh. **c** NAD ^+^ interactions with the ‘closed’ conformation of the WT *Sm*Bdh

Expression of a high-performing BDH enzyme in *Z. mobilis* is critical for 2,3-BDO production in this industrially relevant ethanol producer. Although the structural analysis of *Sm*Bdh was not the primary intent of this study, considering that this enzyme turned out to be the best expressed and most active enzyme in this organism, it was therefore deemed important to determine the reasons behind the improved functionality of this enzyme. The *Z. mobilis* expressed WT *Sm*Bdh crystallized in the space group P4_3_2_1_2 with two protein molecules per asymmetric unit that could be superimposed with r.m.s.d. of 0.271 Å over 1379 atoms. Two molecules found in the asymmetric unit form a tetramer (dimer of dimers) with a symmetry-related dimer (Fig. 2a) with extensive interface surfaces (Table 2, [35]). The tetrameric arrangement is common to the proteins belonging to the SDR superfamily. *Sm*Bdh was shown to be a tetramer in solution by Native PAGE [36]. The main difference between the two molecules in the asymmetric unit is that molecule A is found in ‘closed’ conformation (that could be best described by ~ 10 Å distance between Cα-atoms of residues Ala93 and Trp192) and molecule B is found in ‘open’ conformation (the same distance is ~ 14 Å) (Additional file 4, Fig. 2b and c). Upon examination of the electron density maps, we were able to locate NAD ^+^ cofactor molecule and acetoin molecules in protein molecule A (‘closed’ conformation Fig. 3a), while in molecule B (‘open’ conformation) the corresponding space is occupied by only adenine diphosphate (Fig. 3b).

**Table 2:** Buried area of the protein–protein interfaces in the WT *Sm*BDH tetramer

Assembly	Surface area, Å^2^	Buried area, Å^2^	Buried area, %
ABA^symm^B^symm^	31,520	16,820	53.4
AB^symm^	21,020	3140	14.9
AB	21,530	2640	12.3
AA^symm^	21,150	2830	13.4
BB^symm^	21,940	2410	11.0
A	11,992		
B	12,174

The transition from the ‘open’ conformation into the ‘closed’ seems to be coincidental with NAD ^+^ binding and includes the shift of the loop 183–203, containing two helices, α6 and α7 (Fig. 2b, c). In the process, the short helix α6 is unfolded, but this is compensated by the hydrogen bonds formed between the NAD ^+^ and Thr186Oγ and between the main chain carbonyl of Val184 and Thr189Oγ (Figs. 2c, 3). Additional stabilization is likely provided by the hydrophobic interaction between Met188 side chain and nicotinamide ring (Fig. 2c). Interactions between protein and NAD ^+^ are shown in Fig. 3c.

**Fig. 4:**
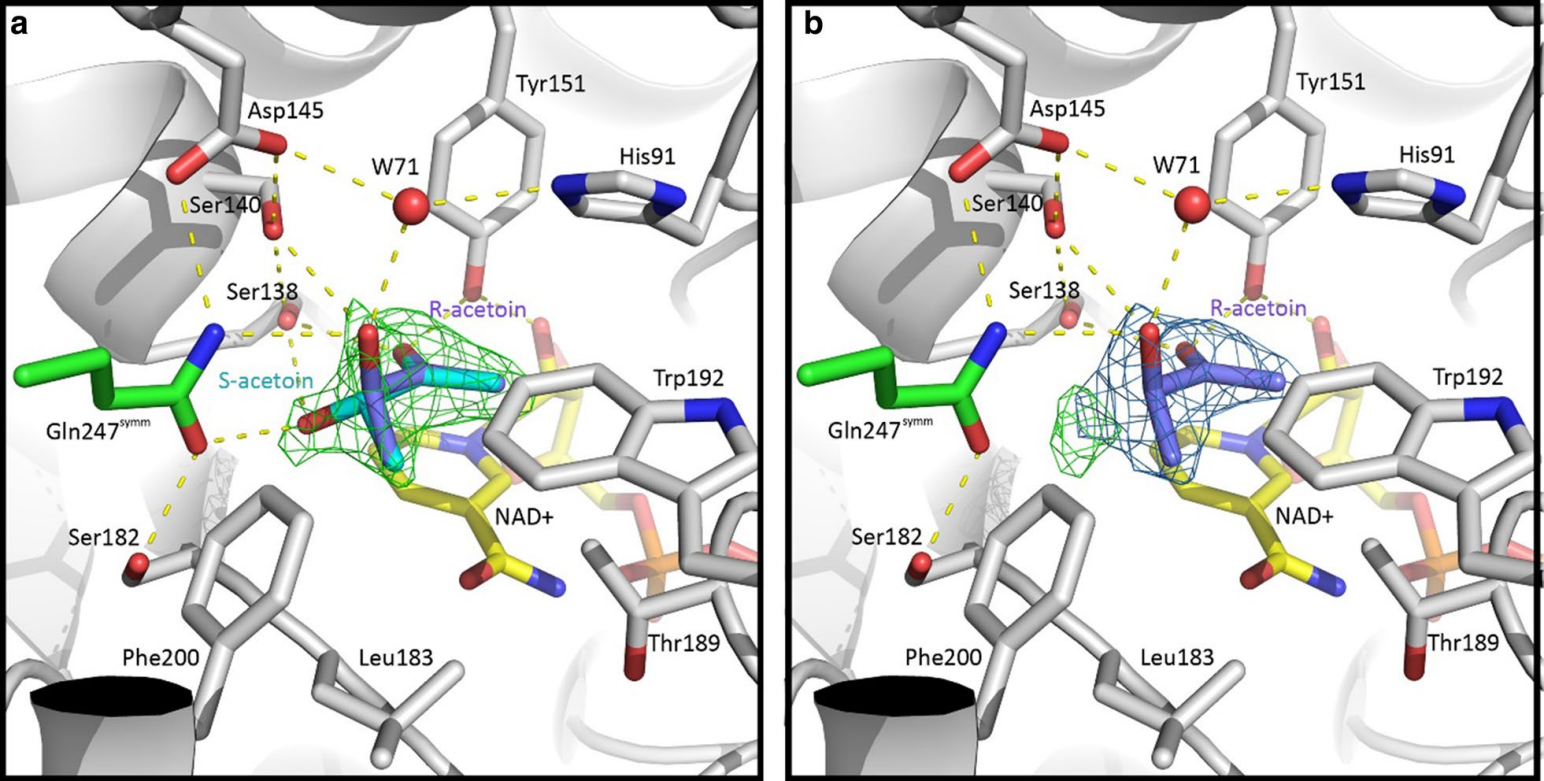
Placement of acetoin molecules in the active site of *Sm*Bdh and its intramolecular interactions. **a** Placement of R- and S-acetoin molecules in the active site of the WT *Sm*BDH. NAD ^+^ (yellow carbons), R- and S-acetoin molecules (purple and cyan carbons), and side chains of surrounding residues (grey carbons) including Gln247 from the symmetry-related molecule (green carbons) are shown in sticks representation. Hydrogen bonds are depicted as yellow dashed lines. Omit Fo-Fc electron density map at 3σ level is shown as a green mesh over acetoin molecules. **b** When only R-acetoin is modeled in the active site of the WT *Sm*BDH, a strong peak in the Fo-Fc map appears after refinement (shown as a green mesh at 3σ level) indicating incomplete interpretation of the electron density maps. 2Fo-Fc map contoured at 1σ level is also shown

WT *Sm*Bdh contains acetoin in the substrate-binding pocket of the ‘closed’ molecule A. Crystallization conditions included NAD ^+^ and acetoin, and more acetoin was used for cryoprotection. Omit electron density maps showed three bulges at the C3 atom (Fig. 4a). When only (3R)-acetoin molecule was modeled, a positive peak at the difference map showed up (Fig. 4b). We interpreted this as both (3R)- and (3S)-acetoin molecules bound in the pocket since commercially available acetoin is a racemic mixture and no other small molecules (like ethylene glycol) were added during crystallization or as a cryoprotectant. Out of three “bulges”, only one should be modeled as a methyl group since its surrounding is hydrophobic (side chains of Leu183, Trp192, Phe200, and Thr189Cγ) and no possible hydrogen bond donors or acceptors are available. Thus, this bulge was modeled as C4 atom of both (3R)-acetoin and (3S)-acetoin. Two other bulges, in contrast, have hydrogen bond partners available: Gln247^symm^Nε (3.1 Å), Ser140Oγ (3.1 Å), or a water molecule (2.7 Å) for (3R)-acetoin O3 atom, and Gln247^symm^Oε (3.0 Å) or Ser138 Oγ (3.2 Å) for (3S)-acetoin O3 atom.

### *Sm*Bdh belongs to the SDR superfamily of enzymes

*Sm*Bdh exhibits the typical single domain short-chain dehydrogenase/reductase (SDR) architecture. A monomer displays a dinucleotide-binding Rossmann fold that includes seven-stranded parallel β-sheet (β3–β2–β1–β4–β5–β6–β7) flanked by three α-helices (α2, α1, and α8) on one side and three α-helices (α3, α4, and α5) on another. There is an additional small lobe on top of a core structure, formed by two short helices, α6 and α7, creating a deep cleft in which the cofactor is bound, and where the active site is located (Figs. 2 and 3). It should be noted that *Sm*Bdh has been shown to be an NADH dependent dehydrogenase enzyme [18]. Two α-turns (αt1 and αt2) can also be recognized in this structure.

PDBeFold [37] search returned over 3000 molecules with Z-score between 6 (usually considered to be the lower threshold for likeliness) and 16.6, and sequence identity up to 37%. Top matching structure could be superimposed over *Sm*Bdh with r.m.s.d. of 1.03 Å over 239 Cα-atoms (FabG from *Bacillus sp.*, PDB ID 4NBU). While SDR superfamily is vast and includes enzymes active towards various substrates, there are several reported Bdh structures, namely from *Burkholderia cenocepacea* (*Bc*Bdh, PDB ID 4WEO), *Burkholderia xenovorans* (*Bx*Bdh, PDB ID 5JY1), *C. glutamicum* (PDB ID 3A28), *Gluconobacter oxydans* (*Go*Bdh, PDB ID 3WTC), and *K. pneumoniae* (*Kp*Bdh, PDB ID 1GEG) that are available in the PDB database. All mentioned enzymes are S-acting or S-installing, introducing 2S chiral center in the acetoin molecule. However, only two enzymes are well characterized with regard to the substrate chiral specificity: Bdh from *K. pneumoniae* (*Kp*Bdh) is (3R)-acetoin dependent [30] and Bdh from *C. glutamicum* (*Cg*Bdh) is (3S)-acetoin dependent [31]. An explanation for the chiral substrate recognition based on the spatial organization of the substrate-binding pocket has been presented before [31].

### Key differences in the *Sm*Bdh structure lead to improved positioning of acetoin in the active site of the enzyme

**Fig. 5:**
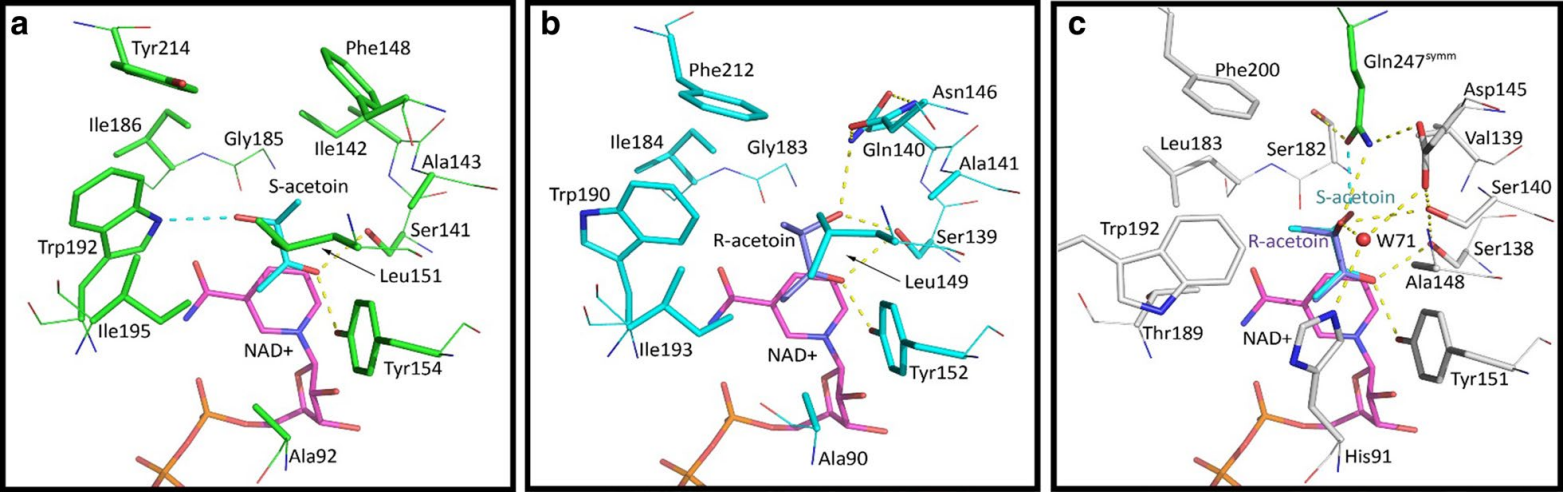
Comparison of the acetoin binding sites of S-acting Bdh enzymes. **a** *Cg*Bdh; **b** *Kp*Bdh, and **c** *Sm*Bdh. Carbon backbone are represented in green for *Cg*Bdh, cyan for *Kp*Bdh and grey for *Sm*Bdh. Main chain atoms of the amino acid residues involved in the substrate binding are shown in lines and side chains are shown in sticks. NAD ^+^ substrate is shown in sticks with magenta carbons. (3S)-acetoin in *Cg*Bdh is represented in cyan backbone and (3R)-acetoin in *Kp*Bdh is represented in purple backbone, are modeled following acetoin binging in *Sm*Bdh using C1, C2, C3 and O2 atoms of the acetoin molecules in *Sm*Bdh as anchors. Available hydrogen bonds are depicted as yellow dashed lines. Hydrogen bonds specific for (3S)-acetoin O3 atom are depicted as cyan dashed lines (panels **a** and **c**). W71 indicates a water molecule

Placement of acetoin molecule inside the active site is key to the efficient conversion of this substrate to 2,3-BDO. To determine the structural characteristics that confers this property to the *Sm*Bdh enzyme, we compared this protein with similar Bdhs from other organisms. Based on our assessment, the strategic placement of the hydrophobic cluster on the αt1 side of the acetoin binding site (Ile142, Phe148, Leu151) and possible hydrogen bond partner on the α6 side of the acetoin binding site (Trp192) would be preferential for (3S)-acetoin binding in *Cg*Bdh (Figs. 2b and 5a). The opposite arrangement: hydrophobic cluster on the α6 side of the acetoin binding site (Ile184, Trp190, Ile193, Phe212) and possible hydrogen bond partner(s) on the αt1 side (Ser139, Gln140) would be preferential for (3R)-acetoin binding in *Kp*Bdh (Figs. 2b and 5b). We also analyzed the substrate binding sites of other Bdh enzymes such as those from *Go*Bdh, *Bc*Bdh, and *Bx*Bdh**.** Analyzing substrate binding sites in these three Bdh enzymes suggested that *Go*Bdh and *Bc*Bdh are likely (3S)-acetoin dependent, whereas *Bx*Bdh is likely (3R)-acetoin dependent. For simplicity, we used *Kp*Bdh and *Cg*Bdh as reference structures to compare the acetoin binding site of *Sm*Bdh. *Sm*Bdh stands out when compared to these two protein structures. At a first glance, it looks similar to the *Kp*Bdh—a hydrophobic cluster made of Leu183, Thr189Cγ, Trp192, and Phe200 located on the α6 side of the groove is well suited for interaction with the C4 atom of the acetoin molecule (Figs. 5b and c). However, Gln140 of the *Kp*Bdh is replaced with Val139 in *Sm*Bdh. In this protein, another glutamine side chain makes up for that difference (i.e., Gln247^symm^ from a symmetry-related molecule). Other differences then start to accumulate. Whereas in *Cg*Bdh and *Kp*Bdh the number of partners for possible H-bonds stabilizing O3 atom of acetoin is very limited—one (Trp192Nε) and possibly two (Gln140Nε and Ser139Oγ), respectively, the acetoin binding site in *Sm*Bdh has a vast H-bond network better suited to stabilize both acetoin enantiomers. Specifically, 1: Leu151/149 of *Cg*Bdh/*Kp*Bdh, respectively, is replaced with Ala148 in *Sm*Bdh creating a void where a water molecule W71 is now located (Fig. 5c). This water molecule is held in place by Nε of His91 that replaced Ala 92/90 of *Cg*Bdh/*Kp*Bdh, respectively, and Oδ of Asp145. This water molecule is placed within 2.7 Å from O3 atom of (3R)-acetoin molecule in *Sm*Bdh. 2: Ala143/141 of *Cg*Bdh/*Kp*Bdh, respectively, is replaced with Ser140 in *Sm*Bdh and Ser140Oγ is within 3.1 Å from the O3 atom of (3R)-acetoin. Nε of the Gln247^symm^ is situated 3.1 Å away from the O3 atom of (3R)-acetoin (Fig. 5c). 3: For the (3S)-acetoin, its O3 atom is located 2.8 Å away from Oε of the Gln247^symm^ and 3.2 Å away from Ser138Oγ (Fig. 5c). We can suggest that significant improvements in supporting the hydrogen bond network in the active site of *Sm*Bdh, in comparison to the other known Bdhs, contributes to more precise positioning of acetoin molecule in the active site and probably better stabilization of the reaction intermediate which could in turn lead to higher turnover of the substrate.

### Substrate promiscuity of the *Sm*Bdh enzyme

Most of the characterized Bdh enzymes show strong preference to either (3S)- or (3R)-acetoin as a substrate [24, 33, 38, 39]. *Sm*Bdh was reported to be able to reduce both (3R)- and (3S)-acetoin to 2,3-BDO, although (3R)-acetoin was more readily converted than (3S)-acetoin [18]. On the other hand, *Sm*Bdh was able to oxidize meso-2,3-BDO and (2S,3S)-BDO, while oxidation of (2R,3R)-BDO was not detectable. Moreover, its activity towards (2S,3S)-BDO was only 11% of that towards meso-2,3-BDO [18]. This observation was similar to that reported by Médici et al. where (3R)-acetoin was found to be the preferred substrate in the racemic mixture of acetoin. Fifteen percent of the (3S)-acetoin remained unconverted after 24 h in their study [36]. Indeed, in our crystal structure we were able to locate both acetoin enantiomers in the active site. Whereas the positions of all acetoin atoms, with the exception of O3, superimpose quite well; the O3 atoms of (3S)- and (3R)-acetoin molecules are involved in hydrogen bonds with different surrounding residues (Figs. 4, 5c). This finding supports the idea of productive binding of both acetoin enantiomers in the active site. Still, (3S)- and (3R)-acetoin molecules are not treated equally by the *Sm*Bdh. We suggest that the differences in the hydrogen bond networks of the (3S)-acetoin and (3R)-acetoin can explain our findings. Specifically, the O3 atom of the (3S)-acetoin is only involved in one hydrogen bond with Gln247^symm^ Oε (or Nε if the side chain is flipped). While Ser138Oγ is located only 3.2 Å away from (3S)-acetoin O3, the geometry is not favorable for the H-bond formation. In contrast, O3 atom of the (3R)-acetoin has three possible partners for H-bond formation: water molecule W71 (2.7 Å), Nε of the Gln247^symm^ (3.1 Å), and Ser140Oγ (3.1 Å). It is possible that the "more complex" H-bond network provides better stabilization of the reaction intermediate, which could in turn lead to higher turnover of the (3R)-acetoin in comparison to (3S)-acetoin.

### Shorter α6 helix results in possible improvement in ingress/egress of the cofactor and the substrate/product

Although the general fold of the *Sm*Bdh monomer is very common, we identified several features that are unique to this enzyme. Comparing *Sm*Bdh structure with other Bdh structures we found no coexistence of both ‘open’ and ‘closed’ conformations in other Bdhs: the active sites in all molecules in the tetramer are either equally populated in the liganded structures or not populated at all. In *Sm*Bdh, two molecules of the tetramer are found in an ‘open’ conformation with substrate binding sites empty and two molecules in ‘closed’ conformation with populated substrate binding sites. This observation points to the possibility of a concerted ‘tug-of-war’ motion in the *Sm*Bdh tetramer, where closure of active sites of two diagonally opposite molecules leads to expansion of the active sites of the other two molecules for faster replenishment with the cofactor and the substrate (Fig. 2a).

**Fig. 6:**
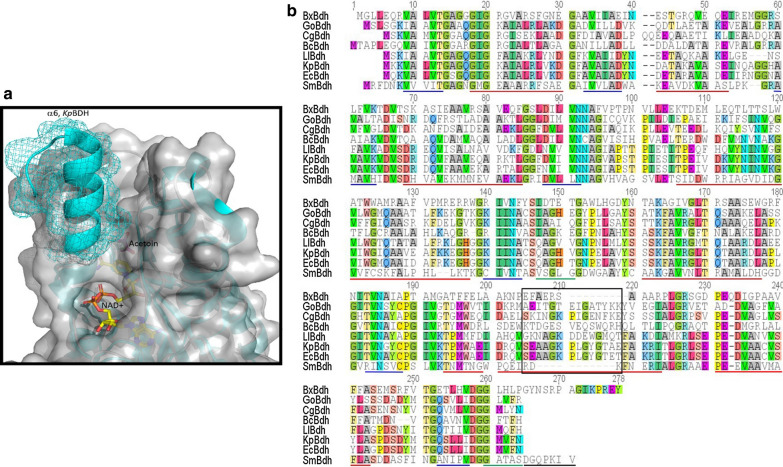
Comparison of different domains of SmBdh with other known Bdh enzymes. **a** Comparison of α6 helix of the *Sm*Bdh and *Kp*Bdh. *Sm*Bdh is shown as grey cartoon covered with semi-transparent surface. NAD ^+^ and acetoin molecules are shown in sticks representation. *Kp*Bdh is shown as cyan cartoon. The larger α6 helix of the *Kp*Bdh is covered in cyan mesh to illustrate the additional volume obstructing the catalytic cleft. **b** Sequence alignment of *Sm*Bdh (accession #AFH00999), *Ec*Bdh (accession #AFM58915), *Kp*Bdh (PDB 1GEG), *Ll*Bdh (UniProtKB—D2BQ24), *Go*Bdh (PDB 3WTC), *Bx*Bdh (PDB 5JY1), *Cg*Bdh (PDB 3A28), *Bc*Bdh (PDB 4WEO) using ClustalW program. The 11 amino acid deletion region in *Sm*BDH is shown with a black box. Red lines, alpha helix; blue lines, beta strand; green lines, alpha turn; black line, C-terminal extension in *Sm*Bdh

Comparison between *Sm*Bdh and Bdhs with known structures shows a shortened α6 helix in *Sm*Bdh (also known as FG1 in SDR structure description) (Fig. 6). The α6 helix is six residues long in *Sm*Bdh, 10 residues long in *Bx*Bdh and 15 residues long in *Kp*Bdh, *Cg*Bdh, and *Go*Bdh. The shortened α6 helix provides wider opening of the catalytic cleft thus improving access to the NADH/NAD + and acetoin/2,3-BDO binding sites and easing ingress/egress of the cofactor and substrate/product.

### Substrate-binding pocket is formed by two protein molecules in *Sm*Bdh, unlike the others

One of the most interesting and exclusive features of *Sm*Bdh is the organization of the active site. We found that the substrate-binding pocket is formed by two protein molecules, not a single peptide as found in all other reported Bdh enzymes. The C-terminus of molecule A protrudes into the groove between α7 helix and the α-turn αt1 capping substrate-binding pocket of molecule A^symm^ and vice versa (Fig. 2a). The side chain of Q247^symm^ is involved in the substrate positioning forming a hydrogen bond with the O3 atom of the acetoin. (Fig. 5). In most SDRs the groove between α7 helix and α-turn αt1 is unobstructed and open to the solvent, but there are examples of active sites with the involvement of the second protein chain, for example: 17β hydroxysteroid dehydrogenase 14 from *Homo sapiens* (1yde, [40, 41]), alcohol dehydrogenase from *Arthrobacter sp*. TS-15 (6qhe, [42]), and glucose dehydrogenase 4 from *Bacillus megaterium* (3auu, [43]). Searching through the PDB, we could not find any other Bdh structure where the active site required the participation of a second protein molecule, so *Sm*Bdh is the first Bdh with this feature. The only similar functional aspect of the involvement of C-terminus in active site formation is found in the case of *Bx*Bdh, where it protrudes into the same protein molecule instead of a different protein molecule, as in *Sm*Bdh.

There are several consequences of this substrate-binding pocket organization. First, *Sm*Bdh would only be active as a tetramer because the active site is incomplete unless tetramerization is achieved. In other Bdh enzymes active sites are solely formed by a single protein molecule and the enzymatic activity in theory could occur in the monomeric or dimeric state. Second, the presence of the C-terminal portion of another protein molecule near the substrate-binding pocket creates a cap making the pocket much less accessible from the solvent compared to other Bdh enzymes. Third, higher specificity towards acetoin is another possible consequence as the substrate binding site is restricted in volume and cannot accept any larger substrate. This concept is in good agreement with the observation that *Sm*Bdh prefers smaller substrates, such as vicinal diketones/diols that do not contain bulky groups [36]. Tighter and more specific binding of the acetoin molecule could be achieved due to an improved hydrogen bonding network (as explained in the section “Key differences in the *Sm*Bdh structure lead to improved positioning of acetoin in the active site of the enzyme”); thereby improving reaction intermediate stabilization and ultimately enhancing catalytic efficiency of the *Sm*Bdh.

### Mutational studies of the *Sm*Bdh

#### Gln247 plays a crucial role in SmBdh catalysis

**Fig. 7:**
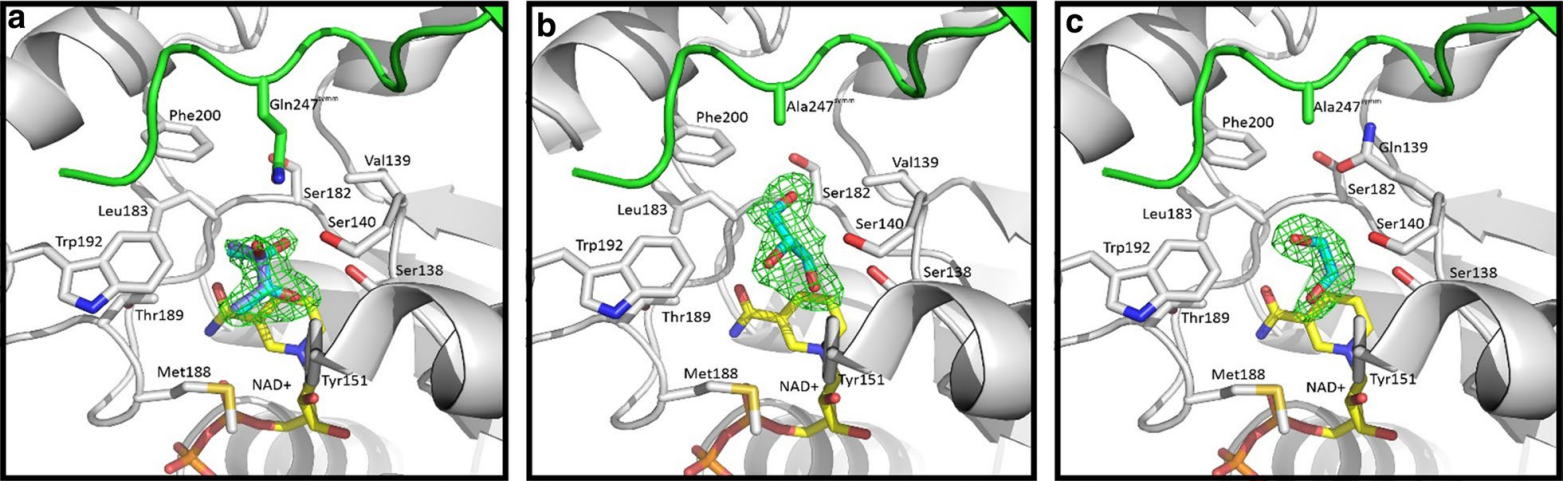
Modeled structures of the active site of *Sm*Bdh and its variants along with their substrates. **a** Omit Fo-Fc electron density map at 3σ level is shown as a green mesh over acetoin molecules in the active site of the WT *Sm*Bdh. **b** Omit Fo-Fc electron density map at 3σ level is shown as a green mesh over glycerol molecule in the active site of the Q247A *Sm*Bdh mutant. **c** Omit Fo-Fc electron density map at 3σ level is shown as a green mesh over ethylene glycol molecule at the active site of the Q247A + V139Q *Sm*Bdh double mutant

Based on structural evidence that we have obtained, Gln247 seems to play an important role in the hydrogen bonding-mediated stabilization of the substrate, as well as better positioning of the substrate in the substrate-binding pocket of the protein. This is important in the context of the overall catalytic ability of this protein; as well as proving to be one of the critical parameters that could make *Sm*Bdh a better performing Bdh enzyme in comparison to its closely related homologs. In order to determine the importance of the Gln247 side chain for the catalysis, we developed two mutants of *Sm*Bdh: (1) Q247A where this side chain is removed, leaving alanine at position 247 and (2) the double mutant Q247A + V139Q, where the missing glutamine side chain would be reinstated at the position 139 that is present in *Kp*Bdh (Fig. 7, Additional files 5 and 6). Whereas Q247A will disrupt the active site of the protein, Q247A + V139Q is expected to restore the active site via a compensatory mechanism resulting in the active site being established by a single protein chain without contribution from a symmetry-related molecule (i.e., from the C-terminus of the opposite molecule in the tetramer).

The *Sm*Bdh Q247A and the Q247A + V139Q mutants crystallized in the same space group (P4_3_2_1_2) as the WT *Sm*Bdh repeating its protein chains arrangement. Two protein molecules per asymmetric unit could be superimposed with r.m.s.d. of 0.275 Å over 1400 atoms (Q247A), and 0.263 Å over 1368 atoms (Q247A + V139Q). As with the WT *Sm*Bdh, the primary difference between the two molecules in the asymmetric unit in the mutant structures was that molecule A was found in ‘closed’ conformation (that could be best described by ~ 10 Å distance between Cα-atoms of residues Ala93 and Trp192) and molecule B was found in ‘open’ conformation (the corresponding distance is ~ 15 Å) (Additional file 4, Fig. 2). Upon examination of the electron density maps, we were able to locate NAD ^+^ cofactor molecule and a substrate in protein molecule A (‘closed’ conformation), whereas in molecule B (‘open’ conformation) the corresponding space is occupied by only adenine diphosphate (as in Q247A mutant) or remains unoccupied (as in Q247A + V139Q mutant) at all. The substrate binding site was found to be occupied by glycerol molecule in Q247A mutant and ethylene glycol in Q247A + V139Q mutant (Fig. 7).

In the Q247A mutant structure, a glycerol molecule was modeled at the substrate-binding pocket (Fig. 7b). Acetoin was present in the crystallization conditions, but our attempts to use additional acetoin for cryoprotection only led to crystal damage. Therefore, a 50/50 v/v mix of glycerol and ethylene glycol was used for cryoprotection. We postulate that the absence of the Gln247^symm^ side chain led to an increase of the available space for the substrate and therefore the larger glycerol molecule could be bound. In the Q247A + V139Q double mutant structure, an ethylene glycol molecule was modeled at the substrate-binding pocket (Fig. 7c). As with the Q247A mutant, acetoin was present during crystallization, but raising its concentration was damaging to the crystals. A 50/50 v/v mix of glycerol and ethylene glycol was therefore used for cryoprotection. We suggest that the available space in the substrate binding site became reduced compared to the Q247A single mutant, thereby providing insufficient space for glycerol to fit. Instead, ethylene glycol which is comparable in size to acetoin could fit.

**Fig. 8:**
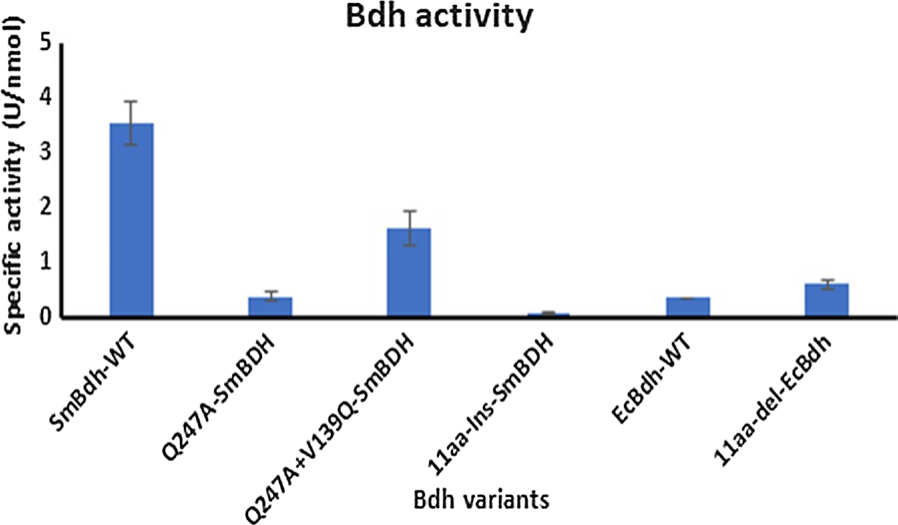
2,3-Butanediol dehydrogenase activity of purified Bdh enzyme variants. Bdh activity was measured by following NADH consumption at 340 nm. Purified proteins were used for the enzyme assay. Reactions were carried out in triplicates and are represented as mean SD

We further tested the activity of both these mutants using the NADH consumption assay to determine the effect of these mutations on the function of the protein. In comparison to the WT *Sm*Bdh, Q247A mutant showed a ~ 90% loss in activity as predicted by the structure, whereas the double mutant Q247A + V139Q showed ~ 300% improvement in activity in comparison to Q247A mutant (Fig. 8). Although the double mutant did not completely restore the loss of Gln247 activity, significant function was regained by introducing the V139Q mutation in this protein. It can thus be inferred that Gln247 is extremely important for the activity of the protein, which cannot be fully restored by a complementary mutation, such as V139Q. However, this is not a thorough analysis of complementation of the Q247A mutation and there could be other mutations that could help restore full functionality of the Q247A mutation. This analysis also highlights the importance of the C-terminal Gln247 from the opposite molecule in conferring full functionality.

### Extending the short α6 helix of *Sm*Bdh results in loss in activity

Comparing the different Bdh enzymes, we found that most of the S-acting Bdh enzymes, such as *Ec*Bdh, *Cg*Bdh, and *Kp*Bdh have longer α6 helix structure (Fig. 6), whereas deletion of 11 amino acid residues in the *Sm*Bdh leads to a shorter α6 helix along with a shorter linker between the α6 and α7 helices. Based on the activity analysis data (Table 1, Fig. 8), it was clear that *Sm*Bdh is much more active than the control enzymes, which include *Ec*Bdh. Furthermore, structural analysis also suggested that the shortened α6 helix contributes to the improved ingress or egress of the substrate and/or the product molecules. This result prompted us to investigate the role of this important structural difference in *Sm*Bdh with regard to enzyme performance. In order to support this hypothesis, we used two Bdh enzymes—*Sm*Bdh with short α6 helix and *Ec*Bdh with long α6 helix. Sequence alignments with highlighted differences are shown in Fig. 6b. We designed two mutants: (1) 11aa-Ins-*Sm*Bdh, where sequence fragment “RDK” was replaced with “SEAAGKPLGYGTET” to mimic longer α6 helix of *Ec*Bdh and (2) 11aa-del-*Ec*Bdh, where the amino acid sequence “SEAAGKPLGYGTET” was replaced with “RDK” to mimic the corresponding shorter α6 helix of *Sm*Bdh. The residues flanking these regions in *Ec*Bdh and *Sm*Bdh showed acceptable homology, so we decided to keep the swap region as small as possible. Proteins were expressed in the *Z. mobilis* 9C strain, purified to homogeneity and subjected to activity analysis using the NADH consumption assay. As expected, activity of the 11aa-Ins-*Sm*Bdh was greatly reduced (down to 3% of the WT activity) with the insertion, whereas the activity of 11aa-del-*Ec*Bdh increased by ~ 70% in comparison to WT *Ec*Bdh (Fig. 8), suggesting that the size of the α6 helix contributes to the unique activity of *Sm*Bdh in comparison to the other S-acting Bdhs. Nevertheless, replacing the longer α6 helix in *Ec*Bdh with the shorter helix from *Sm*Bdh did not render *Ec*Bdh similar to *Sm*Bdh in terms of its activity; we found that the activity of the 11aa-del-*Ec*Bdh was only 17% of WT *Sm*Bdh (Fig. 8). This clearly suggests that while the short α6 helix is important for the high activity of *Sm*Bdh, there are other structural features that this enzyme possesses that also contribute towards its superior performance.

## Conclusions

Based on expression of 11 phylogenetically different Bdh enzymes, we have identified that the *S. marcescens* Bdh enzyme shows the highest activity when expressed in *Z. mobilis*. This enzyme has been classified as an S-acting Bdh based on production of meso-2,3-BDO and (2S,3S)-BDO from a racemic mixture of acetoin. We have structurally characterized this enzyme and ascertained the distinct structural features that may be critical for its activity. Specifically: (1) this enzyme is organized as a tetramer with diagonally opposite protein molecules acting in tandem, such that one diagonally opposite pair is bound by the substrate in the closed conformation, with the other two protein molecules in the tetramer found ready to take up new substrate molecules in the open confirmation. This is the first instance of a piston-type function of a Bdh enzyme. (2) The active site of the *Sm*Bdh enzyme is formed with the participation of the Gln247 of the opposite molecule from the tetramer, removal of this residue causes severe functional defects to the protein. A complementary mutation that introduces a glutamine partially restores the function of this protein. (3) *Sm*Bdh possesses improved hydrogen bond network resulting in better positioning of the substrate in its active site. (4) *Sm*Bdh is able to bind to both (3S)- and (3R)-acetoin productively and we were able to locate both substrate molecules in the active site of the crystal structure, thus providing the structural confirmation to the enzyme substrate promiscuity. (5) The presence of a shorter α6 helix provides a wider cleft for efficient entry and exit of the substrate and products, respectively, to *Sm*Bdh. We have experimentally verified this hypothesis by introducing residues from a low activity Bdh enzyme (i.e., *Ec*Bdh), which resulted in dramatic reduction in activity of this protein. (6) Finally, we have also demonstrated that deletion of residues that shortened the α6 helix of *Ec*Bdh can result in dramatic increase in its activity (~ 70%), which could prove to be a game-changer when considering low-active Bdh enzymes for 2,3-BDO production. Overall, this work has identified a superior heterologous Bdh enzyme suitable for expression in *Z. mobilis*. Moreover, availability of a strain expressing this Bdh enzyme is expected to greatly alleviate one of the major bottlenecks involved in 2,3-BDO production by this organism. The next logical step would be to incorporate the *Sm*BDH into a *Z. mobilis* strain carrying the other two BDO pathway genes, *Als* and *Aldc*. Our future work will involve testing the *Sm*Bdh in combination with other Als and Aldc enzymes to identify the best combination of enzymes that would lead to improvement in 2,3-BDO production.

## Materials and methods

### Strain and growth conditions

*Zymomonas mobilis* strain 9C (ZM4 derivative strain with xylose-utilizing abilities) was revived from glycerol stocks and grown in rich medium (RM, 10 g/L yeast extract, 2 g/L KH_2_PO_4_) containing 5% glucose (RMG) at 30 °C under shaking conditions (120 rpm). Spectinomycin at a concentration of 200 µg/mL was used to maintain transformant colonies.

### Phylogenetic analysis of Bdh

Initially, 52 Bdh amino acid sequences were obtained from the NCBI database. These Bdh sequences represented one enzyme per genus under the assumption that Bdh enzymes within the same genus would have similar structures and functions. Two distinct sets of Bdh sequences were formed based on their sequence lengths. One set contained ~ 350 aa, while the other contained ~ 250 aa. In genera containing both sequence types, both the sequences were selected. In the end, 57 Bdh sequences were used for phylogenetic analysis. Full-length Bdh sequences were aligned using ClustalW, and phylogenetic analysis was carried out using the MEGA X software [44]. The unrooted evolutionary tree was generated using the neighbor joining method [45]. A bootstrap value of 1000 replications was used for the analysis [46]. All gaps and ambiguous positions were removed using the pairwise deletion option. The evolutionary distances were calculated using the Poisson correction method [47].

### Plasmid construction, transformation, and screening of transformants

Eleven *bdh* gene sequences were codon-optimized for expression in *Z. mobilis* (Additional file 2), synthesized and cloned into the vector pEZ15Asp [15] under the control of the pyruvate decarboxylase promoter (PDC) by GenScript (Piscataway, NJ, USA). These included *Erwinia amylovora *(*Ea*), *Myroides odoratimimus* (*Ma*), *Staphylococcus warneri* (*Sw*), *Thermococcus gammatolerans* (*Tg*), *A. vinelandii* (*Av*), *Mycobacterium Smegmatis* (*Ms*), *Micrococcus luteus* (*Ml*), *A. tumefaciens* (*At*), *S. marcescens* (*Sm*), *Streptomyces coelicolor* (*Sc*), and *Dickeya dadantii* (*Dd*). Prior to transformation, these plasmids were transformed into a Dam- Dcm- *E. coli* strain to avoid methylation to the DNA. These plasmids were transformed into the *Z. mobilis* 9C strain using the protocol described in Yang et al. [15]. Briefly, 1 µg of plasmid DNA was mixed with 50 µL of freshly thawed competent cells in a 0.1 cm gap electroporation cuvette on ice. Cells were electroporated using a Bio-Rad Gene Pulser (Bio-Rad Laboratories, Inc. Hercules, CA, USA) using the following settings: 200 Ω, 25 µF, 1.6 kV. The tubes were immediately transferred to ice for cooling. One milliliter of mating medium (MM; 50 g/L glucose, 10 g/L yeast extract, 5 g/L tryptone, 2.5 g/L (NH_4_)_2_SO_4_, and 0.2 g/L K_2_HPO_4_ containing 1 mM MgSO_4_ was added to the tube, the liquid transferred to a 1 mL cryovial and then incubated for 5–6 h at 30 °C to allow cell recovery. Cells were then plated on MM agar containing spectinomycin (200 µg/mL) and incubated in an anaerobic jar (containing a gas pak) at 30 °C for 2 days. Individual colonies were restreaked on MM medium containing spectinomycin followed by colony PCR to check for the presence of the *bdh* gene. Colony PCR was carried out using the forward primer, SV-90, and the reverse primer, SV-91. All primers used in this study are available in Additional file 7. These primers flanked the promoter and terminator sequences, respectively, thereby amplifying the complete gene cassette.

### Construction of Bdh variants for crystal structure assessment

Two different primers SV-161 and SV-162 were used to amplify the pEZ15Asp plasmid containing the *Smbdh* gene, such that a 6X-histidine tag and a Tobacco etch virus protease cleavage site (ENLYFQG) sequences were introduced, in that order, immediately following the start codon of the *Smbdh* gene. The PCR product was subjected to *Dpn*I digestion followed by ligation using the In-Fusion Cloning protocol (Takara Bio USA, Inc., Mountain View, CA, USA) to obtain the final plasmid, which will be henceforth referred to as “N-term *Sm*Bdh”.

The Q247A *Sm*Bdh mutant was generated by amplifying the N-terminal His-TEV-*Smbdh* containing vector with primers SV-274 and SV-275 to introduce the Q247A mutation within the *bdh* coding fragment, while amplifying the entire plasmid. This PCR product was then subjected to *Dpn*I treatment followed by recircularization using the In-Fusion Cloning protocol to obtain the final pla*s*mid.

The double mutant, Q247A + V139Q *Sm*Bdh, was generated by amplifying the Q247A *Smbdh* pla*s*mid with primers SV-279 and SV-280. Following *Dpn*I treatment, the PCR product was self-ligated using the In-Fusion Cloning protocol to obtain the final plasmid.

The 11aa-Ins-*Sm*Bdh mutant was constructed by amplifying the N-term *Sm*Bdh plasmid with primers SV-277 and SV-276 to introduce the *Enterobacter cloacae* Bdh (*Ec*Bdh) 14 amino acid coding sequence “SEAAGKPLGYGTET” replacing 3 amino acids from *Sm*Bdh (“RDK”) into the *Smbdh* gene along with amplification of the entire vector. This PCR product was subjected to *Dpn*I treatment followed by self-ligation using the In-Fusion Cloning protocol. Prior to cloning the 14 amino acid sequence, the respective *E cloacae* sequence was codon-optimized for expression in *Z. mobilis*.

In order to introduce a 6X-histidine tag and a TEV protease cleavage site into the N-terminal end of the *E. cloacae* Bdh (N-term *Ec*Bdh), the untagged version of the *Ecbdh* gene was first amplified from a previously transformed *Z. mobilis* strain BC-11B [15] using the primers SV-285 and SV-286. The vector fragment was obtained by amplifying pEZ15Asp-*Smbdh* vector with primers SV-283 and SV-284 followed by *Dpn*I treatment of the PCR product. Both these fragments were ligated using the In-Fusion Cloning protocol to obtain the *Ecbdh* gene in the same expression vector, pEZ15Asp. The N-terminal histidine tag along with the TEV site was then introduced by amplifying the pEZ-15Asp-*Ecbdh* vector with primers SV-293 and SV-294, followed by *Dpn*I treatment of the PCR product and self-ligation by the In-Fusion Cloning protocol.

The 11 amino acid deleted version of the *Ecbdh* gene (11aa-del-*Ec*Bdh) was cloned by amplifying the N-term *Ecbdh* plasmid with primers SV-302 and SV-303 to amplify the entire plasmid, except the 14 amino acid sequence (“SEAAGKPLGYGTET”) of *Ec*Bdh, while replacing this segment with a 3 amino acid sequence (“RDK”) of *Sm*Bdh. Following amplification of the entire plasmid, this PCR product was subjected to *Dpn*I treatment followed by circularization using the In-Fusion Cloning protocol.

### Bdh protein expression and purification

Following confirmation of the strains for the presence of the *bdh* genes, individual colonies were inoculated into 5 mL RMG medium containing 200 µg/mL spectinomycin and incubated at 30 °C for 2 days under shaking conditions (120 rpm). An aliquot of this pre-grown culture was then transferred to 20 mL RMG medium containing 200 µg/mL spectinomycin for small scale protein extraction and activity analysis. For large-scale protein purification studies, 750 mL cultures were started in duplicates. In both cases, the starting OD_600_ of the cultures were adjusted to 0.025. Cultures were incubated at 120 rpm at 30 °C for 3 days. The final OD_600_ of the cultures were between 3 and 6 for the different cultures. Cultures were then transferred to ice prior to centrifugation at 6000 × *g* for 5 min to obtain cell pellets. Cells were immediately frozen in liquid nitrogen prior to further processing.

For small-scale protein extraction, cells were thawed on ice and resuspended on 1 mL of 100 mM phosphate buffer (pH 7.0) containing protease inhibitor cocktail (Sigma-Aldrich Corp. St. Louis, MO, USA). Cells were disrupted by sonication using four cycles of 30 s pulses, with intermittent cooling on ice for 30 s between cycles. Cells were then subjected to bead beating for 10 min at 4 °C to ensure complete lysis of the cells. Protein extract was clarified by centrifugation at 13,000 × *g* for 10 min. Total protein content was estimated using the Bradford reagent protocol (Bio-Rad Laboratories, Inc. Hercules, CA, USA).

For large-scale protein extraction, frozen cell pellets were thawed and resuspended at room temperature with equal volume of buffer (50 mM Tris pH 7.5, 100 mM NaCl and 10 mM imidazole) and lysed using lysozyme and vortexing with glass beads. Specifically, 1 mg/mL lysozyme (Hampton Research, Aliso Viejo, CA, USA), 1 U/mL Pierce Universal Nuclease (Thermo Scientific, Rockford, IL, USA) and EDTA-free protease inhibitor (Thermo Scientific, Rockford, IL,USA according to manufacturer instructions) were added and the lysis mixture was incubated for 30 min at room temperature. Lysis was completed via vortexing with glass beads (0.1 mm diameter, 1:1 volume with the cell pellet) for 3–5 min and cell debris was removed by centrifugation for 15 min at 22,000 × *g*. The resulting supernatant was loaded into a 5-mL HisTrap FF crude column (GE Life Sciences, Piscataway, NJ, USA) using an Akta FPLC system (GE Life Sciences, Piscataway, NJ, USA) in 50 mM Tris pH 7.5, 100 mM NaCl and 10 mM imidazole. After loading and washing the unbound proteins from the column, the target proteins were eluted using 50 mM Tris pH 7.5, 100 mM NaCl and 250 mM imidazole. Minor impurities were removed by size exclusion chromatography using HiLoad Superdex 75 (26/60) (GE Life Sciences, Piscataway, NJ, USA) in 20 mM Tris pH 7.5 and 100 mM NaCl. Peaks corresponding to the Bdh samples were pooled and concentrated to 5–15 mg/mL. Protein concentration was measured using absorbance at 280 nm in a NanoDrop1000 and the protein-specific extinction coefficients and molecular weights.

### Protein crystallization

Initial screening was done in a sitting drop vapor diffusion setup using a 96-well plate with Crystal Screen HT, PEG/Ion HT and Grid Screen Salt HT from Hampton Research (Aliso Viejo, CA, USA). Fifty microlitre of well solution was added to the reservoir with three drops made of 0.2 µL of well solution and 0.1/0.2/0.3 µL of protein solution using a Phoenix crystallization robot (Art Robbins Instruments, Sunnyvale, CA, USA). The final crystals for all three structures were grown at 20 °C using an optimization screen with 0.1 M sodium malonate pH 6–7 and 6–15% w/v polyethylene glycol 3350 as the well solution. The protein solution for WT *Sm*Bdh contained 9 mg/mL of protein, 20 mM Tris pH 7.5, 100 mM NaCl, 20 mM NAD^+^ and 200 mM acetoin. *Sm*Bdh Q247A protein solution consisted of 4.2 mg/mL of protein, 20 mM Tris pH 7.5, 100 mM NaCl, 20 mM NAD^+^ and 20 mM acetoin. *Sm*Bdh Q247V + V139Q had 14.5 mg/mL of protein, 20 mM Tris pH 7.5, 100 mM NaCl, 20 mM NAD^+^ and 20 mM acetoin.

### Data collection and processing

Before data collection the *Sm*Bdh crystals were flash frozen in a nitrogen gas stream at 100 K followed by data collection using an in-house Bruker X8 MicroStar X-Ray generator with Helios mirrors and a Bruker Platinum 135 CCD detector (Bruker AXS LLC, Madison, WI, USA. *Sm*Bdh Q247A and *Sm*Bdh Q247V + V139Q crystals were further protected from ice formation by shortly moving them into a well solution drop with 15% (v/v) ethylene glycol and 15% (v/v) glycerol for improved cryo protection. Bruker Suite of programs version 2014-9 (Bruker AXS LLC, Madison, WI, USA) was used for data indexing and processing.

### Structure solution and refinement

The CCP4 package of programs [48] was used for converting intensities into structure factors, for project tracking, and access to the individual programs. Five percent of reflections were reserved for R_free_ calculations using programs F2MTZ, Truncate, CAD and Unique. The structure of the WT *Sm*Bdh was determined via molecular replacement using MOLREP [49]. The initial model was built based on PDB entry 4ni5 with FFAS03 search and ProtMod modeling servers [50–52] using the SCWRL method. The resulting model was refined in REFMAC5 [53] version 5.8.0258 and rebuilt and inspected using Coot version 0.8.0.2 [54]. Structures of the *Sm*Bdh Q247A mutant and Q247A + V139Q double mutant were determined via molecular replacement as described above using the WT *Sm*Bdh as a model. Ramachandran plot was calculated with MOLPROBITY [55] and root mean square deviations (r.m.s.d.) of bond lengths and angles were calculated using ideal values of Engh and Huber stereochemical parameters [56]. Wilson B-factor was obtained from CTRUNCATE [48] version 1.0.11. All structures have been deposited to the Protein Data Bank with PDB codes 6XEW (WT *Sm*Bdh), 6VSP (*Sm*Bdh Q247A), and 6XEX (*Sm*Bdh Q247A + V139Q). Data collection and refinement statistics are shown in Table 3.

**Table 3:** X-ray data collection and refinement statistics

	WT *Sm*BDH	*Sm*BDH Q247A	*Sm*BDH Q247A + V139Q
Data collection			
Space group	P4_3_2_1_2	P4_3_2_1_2	P4_3_2_1_2
Unit cell (Å, °)	*a* = *b* = 108.17, *c* = 82.88 *α* = *β* = *γ* = 90.00	*a* = *b* = 108.68, *c* = 82.95 *α* = *β* = *γ* = 90.0	*a* = *b* = 108.80, *c* = 83.11 *α* = *β* = *γ* = 90.0
Wavelength (Å)	1.54178	1.54178	1.54178
Temperature (K)	100	100	100
Resolution (Å)	25.0–2.0 (2.1–2.0)	25.0–1.9 (2.0–1.9)	25.0–1.8 (1.9–1.8)
Unique reflections	33,823 (4496)	39,724 (5531)	46,796 (6911)
*R*_int_^a^	0.138 (0.689)	0.109 (0.748)	0.093 (0.793)
Average redundancy	11.4 (8.8)	17.3 (10.6)	17.0 (12.5)
< I > / < σ(I) >	11.2 (2.0)	18.7 (2.6)	21.7 (1.9)
Completeness (%)	100 (100)	100 (100)	100 (100)
Refinement			
*R*/*R*_free_	0.162 (0.214)/0.279 (0.378)	0.168 (0.340)/0.222 (0.347)	0.160 (0.210)/0.295 (0.342)
Protein atoms	3736	3792	3766
Water molecules	432	551	558
Other atoms	141	151	70
r.m.s.d. from ideal bond length^b^ (Å)	0.010	0.009	0.010
r.m.s.d. from ideal bond angles^b^ (°)	1.497	1.486	1.612
Wilson B-factor	21.6	19.5	21.2
Average B-factor for protein atoms (Å^2^)	25.2	25.0	24.0
Average B-factor for water molecules (Å^2^)	33.0	32.6	33.1
Ramachandran plot statistics^c^ (%)			
Allowed	99.8	99.8	99.8
Favored	96.9	97.2	97.3
Number of outliers	1	1	1

Statistics for the highest resolution bin are shown in parenthesis

^a^*R*_int_ = ∑hkl ∑i|Ii(hkl)—‹I(hkl)›|/ ∑hkl ∑i Ii(hkl), where Ii(hkl) is the intensity of an individual reflection and ‹I(hkl)› is the mean intensity of a group of equivalents; the sums are calculated over all reflections with more than one equivalent measured

^b^[56]

^c^[55]

### Bdh activity analysis

Bdh activity was assayed by following NADH oxidation at 340 nm for 5 min using a molar extinction coefficient of 6.22 mM^−1^ cm^−1^. The enzyme reaction was carried out in a total volume of 200 µL and contained 100 mM potassium phosphate buffer (pH 7.0), 0.25 mM NADH and 25 mM acetoin. The reaction was started by addition of the enzyme. All reactions were carried out in microtiter plates and monitored using a FLUOstar Omega plate reader (BMG Labtech GmbH, Ortenburg, Germany). Enzyme activities were represented as unit per nmol of the purified protein. One unit of enzyme was defined as the amount of enzyme that consumed one micromole of NADH per minute. For total proteins, enzyme activities were represented as micromole of NADH consumed per min per mg of total protein.

For phylogenetic screening of the Bdh enzymes, whole cell extracts obtained from the small-scale extraction procedure were used for enzyme assays. A range of concentrations from 1–20 µg total protein were tested for each protein extract. For the Bdh assays involving purified proteins (*Sm*Bdh and *Ec*Bdh), a range of protein concentrations from 0.05–1 µg were used.

## Supplementary information

**Additional file 1.** PCR analysis of *Z. mobilis* tranformants containing the different Bdh genes. Eleven Bdh genes were transformed into *Z. mobilis* strain 9C and plated on RMG medium containing spectinomycin. Eight independent colonies were selected for colony PCR analysis using gene specific primers. Five microliters of the PCR products were run on 1% agarose gel and visualized using FluoChem Gel analyzer. White bar represents sets of 8 lanes for each Bdh gene. Av, *Azotobacter vinelandii*; Ml, *Micrococcus luteus;* Ea, *Erwinia amylovora;* Sw, *Staphylococcus warneri*; Sc, *Streptomyces coelicolor*; Sm, *Serratia marcescens*; Dd, *Dickeya dadantii*; Tg, *Thermococcus gammatolerans*; At,* Agrobacterium tumefaciens*; Ms, *Mycobacterium Smegmatis*; Mo, *Myroides odoratimimus*; 9c, *Z. mobilis* control strain.

**Additional file 2.** BDH genes selected for expression in *Z. mobilis*.

**Additional file 3.** Detection of heterologous Bdh proteins from *Z. mobilis* transformants using total protein staining. Five independent transformant colonies were selected for protein extraction. Total proteins were separated on 4-12% SDS PAGE gel followed by Coomassie staining. Arrows represent the expected molecular weight of the individual Bdh proteins. Two Bdh protein sets are shown in each PAGE gel. Each protein set is represented by 5 lanes as indicated by the black bars. Bdh protein names are shown above the black bars. Av, *Azotobacter vinelandii*; Ml, *Micrococcus luteus;* Ea, *Erwinia amylovora;* Sw, *Staphylococcus warneri*; Sc, *Streptomyces coelicolor*; Sm, *Serratia marcescens*; Dd, *Dickeya dadantii*; Tg, *Thermococcus gammatolerans*; At,* Agrobacterium tumefaciens*; Ms, *Mycobacterium Smegmatis*; Mo, *Myroides odoratimimus*; C, *Z. mobilis* control strain.

**Additional file 4.** Ala93Cα-Trp192Cα distance in ‘open’ and ‘closed’ molecules, Å.

**Additional file 5.** Multiple alignment of N-terminal histidine tagged *Sm*Bdh sequences representing the sites of amino acid changes. Three different versions of *Sm*Bdh are shown. Boxes indicate the amino acids that were replaced in the other mutants. *Sm*Bdh (N-term), wild-type; Q247A-*Sm*Bdh (N-term) and Q247A+V139Q-*Sm*Bdh (N-term), two different variants of *Sm*Bdh.

**Additional file 6.** 2,3-butanediol dehydrogenase gene sequences used in the study.

**Additional file 7.** Primers used in this study.

## Data Availability

All data generated or analyzed during this study are included in this published article and its supplementary information files.

## Abbreviations

2,3-BDO: 2,3-butanediol
Bdh: 2,3-butanediol dehydrogenase
NAD: Nicotinamide adenine dinucleotide (oxidized)
NADH: Nicotinamide adenine dinucleotide (reduced)
Als: Acetolactate synthase
Ndh: NADH oxidase
PDB: Protein data bank
SDR: Short-chain dehydrogenase/reductase
PCR: Polymerase chain reaction
α6: Alpha 6 helix
*Kp*Bdh: *Klebsiella pneumoniae* Bdh
*Go*Bdh: *Gluconobacter oxydans* Bdh
*Bc*Bdh: *Burkholderia cenocepacea* Bdh
*Bx*Bdh: *Burkholderia xenovorans* Bdh
*Cg*Bdh: *Corynebacterium glutamicum* Bdh
*Sm*Bdh: *Serratia marcescens* Bdh
*Ec*Bdh: *Enterobacter cloacae* Bdh
*Ea*: *Erwinia amylovora*
*Ma*: *Myroides odoratimimus*
*Sw*: *Staphylococcus warneri*
*Tg*: *Thermococcus gammatolerans*
*Av*: *Azotobacter vinelandii*
*Ms*: *Mycobacterium smegmatis*
*Ml*: *Micrococcus luteus*
*At*: *Agrobacterium tumefaciens*
*Sc*: *Streptomyces coelicolor*
*Dd*: *Dickeya dadantii*
Gln247: Glutamine 247
r.m.s.d.: Root mean square deviations
Q247A: Glutamine 247 to alanine
V139Q: Valine 139 to glutamine
RMG: Rich medium containing glucose
