# Supplementary material for: Phylogenetics-based identification and characterization of a superior 2,3-butanediol dehydrogenase for Zymomonas mobilis expression
Source: Biotechnol Biofuels. 2020 Nov 10;13:186. doi: 10.1186/s13068-020-01820-x (PMC7656694; doi:10.1186/s13068-020-01820-x)
Supplement: Supplementary file 1 — Additional file 1. PCR analysis of Z. mobilis tranformants containing the different Bdh genes. Eleven Bdh genes were transformed into Z. mobilis strain 9C and plated on RMG medium containing spectinomycin. Eight independent colonies were selected for colony PCR analysis using gene specific primers. Five microliters of the PCR products were run on 1% agarose gel and visualized using FluoChem Gel analyzer. White bar represents sets of 8 lanes for each Bdh gene. Av, Azotobacter vinelandii; Ml, Micrococcus luteus; Ea, Erwinia amylovora; Sw, Staphylococcus warneri; Sc, Streptomyces coelicolor; Sm, Serratia marcescens; Dd, Dickeya dadantii; Tg, Thermococcus gammatolerans; At, Agrobacterium tumefaciens; Ms, Mycobacterium Smegmatis; Mo, Myroides odoratimimus; 9c, Z. mobilis control strain. [file 13068_2020_1820_MOESM1_ESM.docx]

**Additional file 2.** BDH genes selected for expression in *Z. mobilis*

| **BDH gene source** | **Accession Number** | **Expected molecular weight (kDa)** |
| --- | --- | --- |
| *Agrobacterium tumefaciens* | T03816\|Ach5_29300 | 38.05 |
| *Agrobacterium vinelandii* | T00885\|Avin_41760 | 38.13 |
| *Dickeya dadantii* | gi\|307128764 | 26.84 |
| *Erwinia amylovora* | gi\|292486492 | 37.80 |
| *Micrococcus luteus* | T00918\|Mlut_07590 | 37.63 |
| *Mycobacterium smegmatis* | T00434\|MSMEG_5021 | 36.20 |
| *Myroides odoratimimus* | HMPREF9716_02581\|EKB06002 | 38.15 |
| *Serratia marcescens* | gi\|383281417 | 26.22 |
| *Staphylococcus warneri* | T02449\|A284_11700 | 37.24 |
| *Streptomyces coelicolor* | T00085\|SCO0256 | 27.77 |
| *Thermococcus gammatolerans* | T00920\|TGAM_0072 | 38.14 |

The *bdh* gene sources, the respective gene accession IDs, and predicted molecular weights are shown.
