# Supplementary material for: Phylogenetics-based identification and characterization of a superior 2,3-butanediol dehydrogenase for Zymomonas mobilis expression
Source: Biotechnol Biofuels. 2020 Nov 10;13:186. doi: 10.1186/s13068-020-01820-x (PMC7656694; doi:10.1186/s13068-020-01820-x)
Supplement: Supplementary file 3 — Additional file 3. Detection of heterologous Bdh proteins from Z. mobilis transformants using total protein staining. Five independent transformant colonies were selected for protein extraction. Total proteins were separated on 4-12% SDS PAGE gel followed by Coomassie staining. Arrows represent the expected molecular weight of the individual Bdh proteins. Two Bdh protein sets are shown in each PAGE gel. Each protein set is represented by 5 lanes as indicated by the black bars. Bdh protein names are shown above the black bars. Av, Azotobacter vinelandii; Ml, Micrococcus luteus; Ea, Erwinia amylovora; Sw, Staphylococcus warneri; Sc, Streptomyces coelicolor; Sm, Serratia marcescens; Dd, Dickeya dadantii; Tg, Thermococcus gammatolerans; At, Agrobacterium tumefaciens; Ms, Mycobacterium Smegmatis; Mo, Myroides odoratimimus; C, Z. mobilis control strain. [file 13068_2020_1820_MOESM3_ESM.docx]

**Additional file 4.** Ala93Cα-Trp192Cα distance in ‘open’ and ‘closed’ molecules, Å.

| **Structure** | **Molecule A, ‘closed’** | **Molecule B, ‘open’** |
| --- | --- | --- |
| WT | 10.21 | 14.24 |
| Q247A | 9.83 | 15.15 |
| Q247A + V139Q | 9.83 | 14.75 |
