# Supplementary material for: Phylogenetics-based identification and characterization of a superior 2,3-butanediol dehydrogenase for Zymomonas mobilis expression
Source: Biotechnol Biofuels. 2020 Nov 10;13:186. doi: 10.1186/s13068-020-01820-x (PMC7656694; doi:10.1186/s13068-020-01820-x)
Supplement: Supplementary file 4 — Additional file 4. Ala93Cα-Trp192Cα distance in ‘open’ and ‘closed’ molecules, Å. [file 13068_2020_1820_MOESM4_ESM.docx]

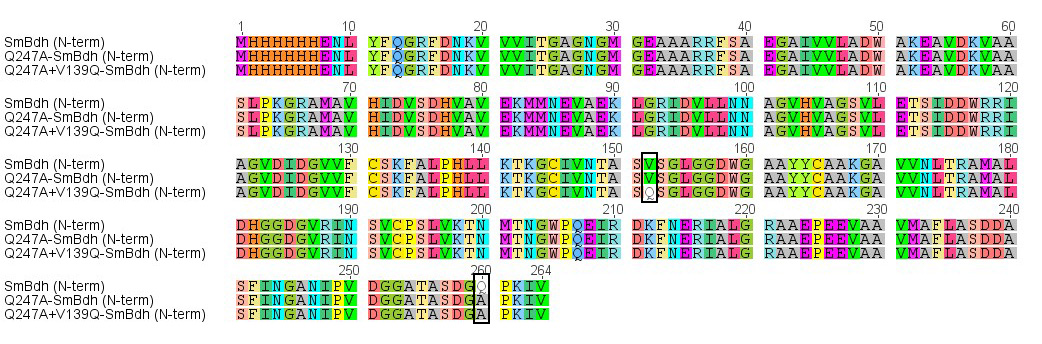


**Additional file 5: Multiple alignment of N-terminal histidine tagged *Sm*Bdh sequences representing the sites of amino acid changes.** Three different version of *Sm*Bdh are shown. Boxes indicate the amino acids that were replaced in the other mutants. *Sm*Bdh (N-term), wild-type; Q247A-*Sm*Bdh (N-term) and Q247A+V139Q-*Sm*Bdh (N-term), two different variants of *Sm*Bdh.
