# Supplementary material for: Phylogenetics-based identification and characterization of a superior 2,3-butanediol dehydrogenase for Zymomonas mobilis expression
Source: Biotechnol Biofuels. 2020 Nov 10;13:186. doi: 10.1186/s13068-020-01820-x (PMC7656694; doi:10.1186/s13068-020-01820-x)
Supplement: Supplementary file 5 — Additional file 5. Multiple alignment of N-terminal histidine tagged SmBdh sequences representing the sites of amino acid changes. Three different versions of SmBdh are shown. Boxes indicate the amino acids that were replaced in the other mutants. SmBdh (N-term), wild-type; Q247A-SmBdh (N-term) and Q247A+V139Q-SmBdh (N-term), two different variants of SmBdh. [file 13068_2020_1820_MOESM5_ESM.docx]

**Additional file 6.** 2,3-butanediol dehydrogenase gene sequences used in the study.

*Erwinia amylovora* *bdh* (codon optimized)

ATGAAAGCCGCTCGTTGGCATAAAGCCCATGATATTCGCATTGAAGATATTGAAGAACCTTTGGTTTCTGCCGGCAAAGTCAAAATTAAAGTTGCTTGGACCGGCATTTGCGGTAGCGATTTGCATGAATATTTGGCCGGCCCGATTTTTGCTCCTGTCGGTAAACCGCATAAATTGTCTCATGATGTCGCCCCTATTGTTATGGGCCATGAATTTAGCGGTAAAGTTGTCGCTGTCGGCAATGGTGTTACCAAAGTCAAAGTTGGCGATCGTGTTGTCGTTGAACCGGTTTTGTCTTGTCGTACCTGCGAAGCCTGTCGCGAAGGCAAATATAATTTGTGTGCTGATTTGGGCTTTCATGGTTTGAGCGGTGGCGGTGGCGGTTTTTCTAGCTTTACCGTCGTTGAAGAACATATGGTTCATCCGATGCCTGAAGGCTTGTCTTATGAACAGGGTGCCTTGGTCGAACCTGCCGCTGTTGCCTTGCATGCTGTCCGTATGAGCAAATTGAAAGCCGGTGATAAAGCCGCTGTCTTTGGCGCTGGTCCGATTGGCTTGTTGATGATTGAAGCCTTGCGCGCCGCTGGTGCTTCTGAAATTTATGTCGTTGAATTGAGCCCTCAGCGTGCCGAAAAAGCTCGCGAATTGGGCGCCAATGTCGTTATTGATCCGTCTCAGCAAGATGCCGTTGCTACCATTCGTGAACGCAGCAAAGGCGGTGTCGATGTTGCTTTTGAAGTCACCGGTGTTCCGGTCGTTTTGAAACAATGCATTGATTCTAGCCGCTATGAAGGCGAAACCATTATTGTTTCTATTTGGGAAGGTGAAGCCAGCTTTCATCCTAATACCGTCGTTTTGGCTGAACGTAATGTTAAAGGCATTATTGCCTATCGCCATATTTTTCCGGCTGTCATGGAATTGATGACCCAGGGTTATTTTCAAGCCGAAAAATTGGTTACCAAACGTATTGATTTGGCTGATTTGGTTACCGAAGGCTTTGAAACCTTGGTCAAAGAAAAGAATCAGGTCAAAATCTTGGTTCGCCCGCCTCAATAA

*Myroides odoratimimus* *bdh* (codon optimized)

ATGAAAACCATGAAAGCCGCTCGTTGGTATGCCGCTAAAGATATTCGCGTTGAAGAAGTCACCATTCCGCAGCCTAAAGCCGGCCAAGTTAAAATTGCCGTCCAGTTTGCTGGTATTTGTGGCTCTGATTTGCATGAATATAATCATGGTCCGCAGTTGATTCCTATGGATGCTCCGTATCCTTTGAATGGTCATCAAGGCGTTACCACCTTGGGCCATGAATTCTCTGGTATCATCGAAGAAGTTGGTGAAGGCGTCACCCATTTTAAAAAAGGCGATCGTGTTGTCGTTGAACCGATCTATAAAAATTTCGATAGCCCTTTCACCACCGCCGGCCAATATAATTTGGGTGAACATTTGGGTTTCATTGGCTTGGCCGGTGATGGTGGCTTTGCTAATTATGTCGTTGTCGAAGAATATATGGTTCATCCGATGCCTACCTCTATGACCTTTGAACAGGGCGCCATGGTCGAACCGGCCGCTGTTGCCGTCTATGCTGTCATGCAGTCTAATATTCAATTGGGTGAAACCGTCTTTATTAGCGGTGCTGGCCCTATTGGCTTGTTGTGTACCCAAGCCGCTTTGGCCGCTGGTGCCAGCACCGTTATTGTCACCGATGTTGCTGAAAAACGTTTGGAAAAAGCCAAAGAAATTGGCGCTACCCATGTCTTTAATGCCATGGATGCTGATTTGAATCAGAAAATCAAAGCCGTTACCGATCAATTGGGTCCGCATGTCTTTTTGGATTGCGCCGGCGTCCAGGCTAGCTATACCACCGGTTTTAATGTTGTCCGCAATGGTGGCACCATTGTTTTGGTCGCCTTGTTTGGCCAGCCTGTTCAACATGATGCTTTGCAGCAAGTCTTGCGTGAAATTACCGTTAAAGGCGTCATTGCTTATCGCAATATTTTTCCGCAGACCATGAAATTGATTTCTAGCGGTCAAATGCCTGTTGAAAAATTGATCACCAATAAAATCTCTTTGGATGAAATCGTCGATCGCGGTTTTGAAGCCTTGATTCAAAATCCGAGCGAAGTTAAAATTTTGATTGATATTGCTAAATAA

*Staphylococcus warneri bdh* (codon optimized)

ATGAAAGCCGCTGTTTGGTATGGTCAGAAAGATGTCCGTGTTGAAGAACGCGAACCGAAAGAATTGCAAGATAATGAAGTCAAAGTTAAAGTCTCTTGGGCCGGTATTTGTGGCACCGATTTGCATGAATATTTGGAAGGTCCGATCTTCATCAGCACCGAAAAACCTGATTTGTTTTTGGGCCAGAAAGCCCCGGTTACCTTGGGTCATGAATTTGCTGGCGTTGTCGAAGAAACCGGTTCTAAAGTTACCAAATTCAATAAAGGCGATCGTGTTGTCGTTAATCCTACCGTCAGCAAACGCGAAAAAGAAGAAAATATCGATTTGTATGATGGTTATTCTTTTATTGGTTTGGGCAGCGATGGTGGCTTTGCCGAATTTACCAATGCTCCGGAAGAAAATGTTTATAAATTGCCTGATAATGTCTCTGATAAAGAAGGCGCCTTGGTTGAACCGACCGCCGTTGCTGTCCAAGCTATTAAAGAAGGTGAAGTTTTGTTTGGCGATACCGTCGCCATTTTTGGTGCTGGCCCTATTGGTTTGTTGACCGTCGTTGCCGCTAAAGCCGCTGGCGCCAGCAAAATCTTCGTCTTCGATTTGTCTGAAGAACGTTTGAGCAAAGCCAAAGCTTTGGGTGCCACCCATGCTATTAATTCTGGCAAAACCGATCCGGTTGATGTCATCAATAAATATACCGAAAATGGTGTTGATGTCAGCTTTGAAGTTGCCGGCGTCGCTCCTACCTTGAAATCTAGCATTGATGTTACCAAAGCCCGCGGTACCGTCGTTATTGTCTCTATTTTTGGCCATCCGATTGAATGGAATCCTATGCAGTTGACCAATACCGGCGTTAAATTGACCAGCACCATTGCTTATACCCCTACCACCTTCCAGCAAACCATCGATTTGATCAATGAAGGCAATTTGAATGTCAAAGATGTCGTTACCGATGAAATCGAATTGGAAAATATCGTTGAATCTGGCTTTGAACAGTTGGTCAATGATAAAAGCCAAGCTAAAATTTTGGTTAAATTGTAA

*Thermococcus gammatolerans* *bdh* (codon optimized)

ATGCATGAAATGTTGGCTGTTCGTTGGCATGGTCGTCGCGATTTGCGCTTGGAAGATATTCCGGAACCTCAGGTTAAACCGGGCTTCATCAAAATCAAAGTCAAAGCCTGTGGTATTTGCGGCACCGATTTGAATGAATATTTGAATGGTCCGATCTTCATCCCTACCGAACGTCCTCATCCGTTAACGGGTCGTACGGCTCCTGTTACCATTGGTCATGAATTTGCTGGCGAAGTTGTCGAAATTGGTGAAGGCGTTAAAGGTTTTGAAGTTGGCGATCGCGTCGCTATTTTTCCTGTCATTCATTGTGGTGAATGCTATTTTTGTCGTCGCGGCATGGAAAATTTGTGTGTTAATTTTGGTGTCACCGGCTTGAGCGAAGATGGTGGCTTTGCCGAATATGCTTTGGTTCGCCCGTATCAAGCCTATAAAATCCCTGAATCTGTTAGCTTCGAAGAAGCCGCTTTGGTCGAACCTTTGAGCGTTGGTGTCCGTGCCGTTAAAAAAGCTGGTTTGTTGCCGGGCGATTCTGTTGTCATTATTGGTGCCGGCCCTATTGGCTTGAGCGTTTTGTTGGTCGCCCGCGCTTCTGGTGCTGGCAAAGTTATTGTTGTCGAACCGTCTCGTGTCCGTCGCAAAAAAGCCTTGGAATTGGGTGCCGATATTGCTATTGATCCTAGCGGCCGTTCTACCGAAGAAGTTGTCGATGAAATTGTTGGTGAAACCGAATTGGGCGCTGATGTTAGCTTTGAATGCGTCGGTTTGAATGAAACCTTTAAAACCGCCGTCGAATCTATTCGTAAAGGTGGCCGCGCTGTTATTTTGGGCGTCTTTAAATGTTTGACCAGCTTTAATGCCAAAGGTTTGGTTGTCGGCGAAAAATCTATTGTTGGTTCTGTCAGCCATTCTGCCGATGATTTTTGCCGTGGCATTAGCTTGATTGCTTCTGGTCGCGTTGATGTCGGCCCGATGATTACCAGCCGTGTCGGTTTGGAAGAAATTATTGAACGCGGCTTTGAAGAATTGGCCAAAAATCGTGAAGGTCATGTTAAAATCTTGGCTGTCGGCAAATAA

*Azotobacter vinelandii* *bdh* (codon optimized)

ATGACCACCTCTCCTGGTAGCACCATGCGTGCCGCTGTTTGGCATGGCCGTCATGATATTCGCGTTGAAAATGTCCCTTTGCCGGCCAATCCGCCTCCGGGTTGGGTTCAGATTCGCGTCGATTGGTGTGGTATTTGCGGCTCTGATTTGCATGAATATGTTGCTGGCCCTGTCTTTATTCCGGTTGAAAAACCTCATCCGTTGACCGGTATTCAGGGCCAATGTATTTTGGGTCATGAATTTTCTGGCGAAATTGTCAAATTGGGTACCGGCGTTAGCGGTTTTGCCCCGGGCGAACGTATTGCCGCTGATGCTTGTCAACATTGCGGTACCTGTTATTATTGCCGCAGCGGCTTGTATAATTTGTGCGAAAAATTGGCCTTTACCGGTTTGATGAATAATGGCGCCTTTGCTGAATTGGTTAATGTCCCTGCCGAATTGTTGTATAAATTGCCTGCTGATTTTCCGGTCGAAGCCGGTGCTTTGATTGAACCGTTGGCCGTCGGCATGCATGCCGTTAAAAAAGCTGGTAATTTGTTGGGCCGTAATGTTGTCGTTGTCGGTGCCGGCACCATTGGTTTGTGTACCATTATGTGCGCTAAAGCCGCTGGCGCCGCTCAGGTTATTGCCTTGGAAATGTCTAGCGCTCGCAAAGCCAAAGCTTTGGAAGTCGGTGCCTCTTTGGTTCTTGATCCGAAAGAATGTGATGCCTTGGCTGAAATTCGTGCCCGCACCAATGGTTTGGGCGCTGATGTCAGCTTTGAATGCATTGGCAATAAACATACCGCCAAATTGGCTATTGATGTTATTCGTAATGCCGGTAAATCTGTTTTGGTCGGCATTTTTGAAGAACCGTCTAGCTTTAATTTCTTTGAATTGGTCAGCACCGAAAAACAAGTTATTGGTGCCTTGGCTTATAATGGCGAATTTGCTGATGTCATTGCCTTTATTGCTGATGGTCGCTTGGATGTTGAACCTTTGATTACCGGTCGTATTGGCTTGGAAGAAATTGTCGGTCGCGGCTTTGAAGAATTGGTTAATAATAAAGAACATAATGTCAAAATCATTGTTTCTCCGCATTAA

*Mycobacterium smegmatis* *bdh* (codon optimized)

ATGAAAGCCGCTGTTTATCATGGTCCGAATAAATTGGAAATTGCCGATTTACCTGAACCTCAGCCTGGTCCTGGTACCGTTAAAGTCAAAGTTGGTTTCAATGGCATCTGCGGTACCGATTTGCATGAATATTATGCCGGTCCGATTTTTGTCCCTACCGAACCGCATCCTTTGACCGGCCAAGTTATGCCGTTGACCATGGGCCATGAATTTGCTGGTACCATTACCGATGTTGGTGCTGGTGTTACGGGTTTTGCTCCTGGTGATCGTGTCGCTATTGAACCTATTTATCGCTGTGGTCATTGCGCTCCTTGTCGTGCCGGCAATTATAATGTTTGCCAGCAAATTGGCTTTCATGGTTTGATGTGTGATGGTGGCATGGCCGAATATACCGTTGTCCCGGTCGATATGTTGCATAAATTGCCTGATAATGTTAGCTTGCAGTTGGGTGCCTTGGTCGAACCGATGTCTGTTGCTTATCATGCCGCTACCTTGGGCGATGTTGGTGCTGGCGATACCGCCGTTGTCTTTGGTGCCGGCCCTATTGGTATTGGCTTGTGGTTTGCTTTGCGCGGTAAAGGCTTGGAAAGCGTCTATGTTGCTGAACCTTCTCCTACCCGTCGCGCTGCTATTGAAGCCTTGGGCGCTCAGACCCTTGATCCGACCCAAGTCGATGTTCCTGCCTTTATTGCTGATGTCACCGGTGGCCGTGGTGCTGATGCCGCTTTTGATGCCGCTGGCGTTGCCCCGGCTATTGAAGCTGCTACGGCTTCTGTTGGTAGCCGTCGCCCTACCATTAGCGTTGCTATTTATGAAAAACCGTTGACCACCCCTTTGTTGAATTTGGTCATGAATGAATCTCGCATTCAAGGCAGCTTGTGTTATACCTCTGCCGATTTTGAAGCCGTTATTGCTTTGATGGCCGATGGCGTCTATGATACCACCGGTTGGGTTACCACCATTGGCATTGATGATGTCATTGATGAAGGTTTTGAAGCTTTGCATGCCGGCCGTAAAATGAAAGTTTTGATTGATCCTGCTATTTAA

*Micrococcus luteus* *bdh* (codon optimized)

ATGCGCGCCGCTCGTTGGCATGGCAAAAAAGATATCCGCATCGAAGAAATCGATTCTCCGACCGCTGGCCCTGGTCAGGTCTTGGTTGATGTCGCCTGGTGTGGCATTTGCGGTACCGATTTGCATGAATATTTGGAAGGTCCGATTTTTATTCCGCCTGCTGGCCATCCGCATCCTATTTCTGGTGATGCCGCTCCTGTTACCTTGGGCCATGAAATGAGCGGTACCGTCGCCGCTTTGGGCGAAGGTGTTACCGATTTGGAAGTCGGCCAGAAAGTTGTCGTTGAACCGTATATTGTTCGCGAAGAAGATCAAGATCGTCCGGATTATAATTTGGCCCCTGATATGAATTTCATTGGTTTGGGTGGCGATGGTGGCGGTTTGGCCGAACAGATTGCTGTTCGTCGCCGTTGGGTTCATCCGGTCGCTGATTCTGTCCCTTTGGATCAAGCCGCTTTGATTGAACCTTTGAGCGTTGCCCATCATGCTTGGGTCCGTGCTGGCTCTCCTACCTCTGGTGTTGCCGTCATTGGCGGTGCTGGCCCTATTGGTGCCTTGACCGCCGCTGTTTTGAAAGGCAAAGGTTTGACCGTTTATGTCTCTGAATTGAGCGAATTGCGCCGTCAGAAAGTCTTGGAAGCTGGCGTCGTTGATGAAGCCTTTGATCCGCGCGAAGTTGATGTCCCTGCTAAAATTCGTGAATTGCATGATGGCCAAGGTGCCGATGTTGGTTTTGAATGTACCTCTGTCGATGTCGTTTTGGATATGTTGTTGGATGCCGTTCGTCCGGGCGGTGTTATTGTCAATGAAAGCATTTGGGGCCATGAACCTGCCGTTGCTTTGCATAAATTGGTCATGAAAGAAATCGATTTGCGCGGCACCATTGCCTATGCTAATGATCATGCTGATACCATTCGTATGGTTGAAGATGGTGCCGTCGATTTGGCTCCGTTTATTACCGGCAAAATTGGTTTGGATGATTTGGTTGATCAGGGTTTCGAAACCTTGATCCATCATAATGAAACCGCCGTTAAAATTTTGGTCAGCCCTACCGGCCAAGGTTTGTAA

*Agrobacterium tumefaciens* *bdh* (codon optimized)

ATGAAAGCCTTGCGTTTTCATGCCGCTAAAGATTTGCGCATTGAAGATGTCGATATGCCGCCTGAACCGGGTTCTGGCGAAGTCTTGGTTGAAAATAAATTCTGTGGTATCTGCGGCACCGATTTGCATGAATATGCTTATGGTCCGATTTTTGTTCCTAAAGAACCGCATCCTTTTACCGGTGCCCATGGCCCGCAGATTTTGGGTCATGAATTTGGTGGCGTTGTCAAAGCTGTCGGTGAAGGCGTCTCTCATGTTAAACCTGGCGATCGTGTTAGCATTCAACCGTTGATTATGCCTCGTCATGGTGATTATTATGCCGATCGCGGCTTGTATCATTTGTCTACCAATTTGGCCTTGGCTGGTTTGTCTTGGCATAGCGGTGGCATGGCCCAGGCCGCTTTGTTGAATGATTATAATGTCCAGCCGATTCCTGATGCTTTGAGCGATCAAGAAGCCGCTTTGATTGAACCGACCGCCGTCGCTGTTTATGCCTGTGATCGTGGTGGCGTCACCGCTGGCTCTAGCGTCTTGGTTACCGGTGCCGGCCCTATTGGTATTTTGGTTGCTATGGCTGCTCGTGCTGCTGGTGCCTCTCAAATTTTTCTTAGCGATTTGAATGATACCCGTTTGGCCTTGGCTCGCGCCGCTTTGGGTGAAGTTCGTACCATTAATCCTAAAACCGAAAAAGTCGGCGATGTTATTCGCGCCGAAACCGAAGGCAATGTCGGCTGTGATGTTGCTATTGAATGCGTCGGTAATGAATATGCCTTGAAAAATTGCGCCGATGCTGTTCGTAAACAGGGCGTTGTCGTTCAAACCGGTTTGCATCCGGGCGAAAATCCTTTGAATTGGTTCGATGTCACCTTCAAAGATATCGATATCCGTGGTTCTTGGGCCTATCCGACCCATTATTGGCCTCGTGTTGCCCGCTTGATTGCTTCTGGCCAGATTCCGGCTAGCCGCATTGTCACCAAAAATGTTTCTTTGAGCGAAGCTGTTACCGAAGGTTTTGATCAATTGCTTGATCCGGCCGGCAAACATTTGAAAATCTTGATCGATTTGAGCCGCTAA

*Streptomyces coelicolor* *bdh* (codon optimized)

ATGACCTCTGTTGATAATGCCACCCCGCGTACGGCTGTTGTTACGGGTGCTGCTCGTGGTATTGGTCGTGGTATTGCCGAACGCTTGGCTGAAGATGGTTTGGATGTTGTCGTTGCCGATTTGCCTTCTATGGCCGAAGAATTGAGCGCTGTTGCCGCTGGCATTGAAAAAACGGGTCGTCGCGCTTTGGCTGTCCATGCTGATGTTACCAGCAAAGAACAGACCGATGCTTTGGTCGCCGCTGCCGCTGATCGTTTTGGCCGCATTGATGTCTATGTTGCCAATGCTGGTATTGCCCGTGTTACCCCGTTGTTGGAAACCGATTTGGATGAATTTGAACAGGTCATGGCTGTTAATTTGCGTGGCGTCTTTTTGTCTTATCAAGCCGCTGCCCGCCAGATGATTGCCCAAGGTGGCGGTGGCAAAATTATTGGCGCTGCCAGCATTGTCGCCCATCGTCCGTTTGCTTTGTTGGGTGCCTATTCTGCTAGCAAATGGGGCGTTCGTGGTTTGACGCAAGCTGCTGCTATGGAATGGGCTCGTCATGGTATTACCGTCAATGCTTATTGTCCTGGCATTGTTGGTACCGATATGTGGGATTTGATTGATGAACGCTTGGCCGAAGAAGCTGGCATTGAAAAAGGTCAAGCCATCAAAAAACATGCCGAATCTATTGCTTTGGGCCGTGTTGAAGAACCTGCCGATGTCGCCGCTTTTGTTTCTTATTTGGCTTCTCGCGATAGCGATTATATGACCGGTCAGAGCGTCATGATTGATGGTGGCATTCAATTTGCTTAA

*Dickeya dadantii* *bdh* (codon optimized)

ATGAAACAGAAAGTTGCCTTGGTCACCGGTGCTGGCCAAGGTATTGGCAAAGCCATTGCTTTGCGTTTGGCCAAAGATGGTTTTGCCGTTGCTGTTGTCGATTATAATAGCGATACCGCCGGCCAGGTCGCTCAAGAAATTCGCCATCATGGTGGCAATGCCATTGCTTTGACCGCTGATGTTTCTAATCGTGATCAGGTTTTTGATGCCGTCCGCACCGCTCATAAACAATTGGGTGGCTTTCATGTTATTGTCAATAATGCCGGTATTGCTCCGACCACCTTGATTGAAGATATTACCCCTGAAATCGTTGATAAAGTCTATAATATCAATGTTAAAGGTGTCATTTGGGGCATTCAGGCCGCTGTTGAAGCCTTTAAAGCTCAGGGTCAAGGTGGCAAAATTATTAATGCCGCTAGCCAAGCCGGTCATGTTGGCAATCCGGAATTGGCCGTCTATTCTAGCTCTAAATTTGCTGTTCGCGGTTTAACGCAGACGGCTGCTCGTGATTTGGCCCCTTTGGGTATTACCGTTAATGCTTATTGTCCGGGCATTGTCAAAACCCCTATGTGGGCCGAAATTGATCGTCAAATTAGCGAAGCCGCTGGTAAACCGTTGGGTTATGGCACCGCCGAATTTGCTAAACGTATTACCTTGGGCCGCTTGTCTGAACCGGAAGATGTTGCCGCTTGCGTCAGCTATTTGGCCGGTCCTGATTCTGATTATATGACCGGCCAGGCTTTGTTGATTGATGGTGGCATGGTTTTTAATTAA

*Serratia marcescens bdh* (*Smbdh*, codon optimized)

ATGCGTTTCGATAATAAAGTTGTCGTTATCACCGGTGCCGGCAATGGTATGGGCGAAGCCGCTGCCCGTCGCTTTTCTGCTGAAGGTGCCATTGTCGTTTTGGCTGATTGGGCCAAAGAAGCTGTCGATAAAGTTGCTGCCTCTTTGCCGAAAGGCCGTGCTATGGCCGTCCATATTGATGTTAGCGATCATGTCGCCGTTGAAAAAATGATGAATGAAGTTGCTGAAAAATTGGGTCGCATTGATGTCTTGTTGAATAATGCCGGTGTCCATGTTGCTGGCTCTGTTTTGGAAACCAGCATTGATGATTGGCGTCGCATTGCCGGTGTCGATATTGATGGCGTCGTCTTTTGTAGCAAATTTGCTTTGCCTCATTTGTTGAAAACCAAAGGCTGCATTGTCAATACCGCCTCTGTTAGCGGTTTGGGTGGCGATTGGGGCGCTGCCTATTATTGTGCTGCCAAAGGTGCTGTCGTTAATTTGACCCGTGCTATGGCCTTGGATCATGGTGGCGATGGCGTCCGCATTAATTCTGTTTGCCCGAGCTTGGTCAAAACCAATATGACCAATGGTTGGCCTCAGGAAATCCGCGATAAATTCAATGAACGTATCGCCTTAGGTCGTGCTGCTGAACCTGAAGAAGTCGCTGCCGTTATGGCCTTTTTGGCTTCTGATGATGCCAGCTTTATTAATGGTGCTAATATTCCGGTTGATGGTGGCGCTACCGCCTCTGATGGCCAACCTAAAATTGTTTAA

**Sequences for mutation studies**

N-term *Smbdh*

ATGCATCACCATCATCATCATGAGAATCTGTACTTTCAGGGTCGTTTCGATAATAAAGTTGTCGTTATCACCGGTGCCGGCAATGGTATGGGCGAAGCCGCTGCCCGTCGCTTTTCTGCTGAAGGTGCCATTGTCGTTTTGGCTGATTGGGCCAAAGAAGCTGTCGATAAAGTTGCTGCCTCTTTGCCGAAAGGCCGTGCTATGGCCGTCCATATTGATGTTAGCGATCATGTCGCCGTTGAAAAAATGATGAATGAAGTTGCTGAAAAATTGGGTCGCATTGATGTCTTGTTGAATAATGCCGGTGTCCATGTTGCTGGCTCTGTTTTGGAAACCAGCATTGATGATTGGCGTCGCATTGCCGGTGTCGATATTGATGGCGTCGTCTTTTGTAGCAAATTTGCTTTGCCTCATTTGTTGAAAACCAAAGGCTGCATTGTCAATACCGCCTCTGTTAGCGGTTTGGGTGGCGATTGGGGCGCTGCCTATTATTGTGCTGCCAAAGGTGCTGTCGTTAATTTGACCCGTGCTATGGCCTTGGATCATGGTGGCGATGGCGTCCGCATTAATTCTGTTTGCCCGAGCTTGGTCAAAACCAATATGACCAATGGTTGGCCTCAGGAAATCCGCGATAAATTCAATGAACGTATCGCCTTAGGTCGTGCTGCTGAACCTGAAGAAGTCGCTGCCGTTATGGCCTTTTTGGCTTCTGATGATGCCAGCTTTATTAATGGTGCTAATATTCCGGTTGATGGTGGCGCTACCGCCTCTGATGGCCAACCTAAAATTGTTTAA

Q247A *Smbdh*

ATGCATCACCATCATCATCATGAGAATCTGTACTTTCAGGGTCGTTTCGATAATAAAGTTGTCGTTATCACCGGTGCCGGCAATGGTATGGGCGAAGCCGCTGCCCGTCGCTTTTCTGCTGAAGGTGCCATTGTCGTTTTGGCTGATTGGGCCAAAGAAGCTGTCGATAAAGTTGCTGCCTCTTTGCCGAAAGGCCGTGCTATGGCCGTCCATATTGATGTTAGCGATCATGTCGCCGTTGAAAAAATGATGAATGAAGTTGCTGAAAAATTGGGTCGCATTGATGTCTTGTTGAATAATGCCGGTGTCCATGTTGCTGGCTCTGTTTTGGAAACCAGCATTGATGATTGGCGTCGCATTGCCGGTGTCGATATTGATGGCGTCGTCTTTTGTAGCAAATTTGCTTTGCCTCATTTGTTGAAAACCAAAGGCTGCATTGTCAATACCGCCTCTGTTAGCGGTTTGGGTGGCGATTGGGGCGCTGCCTATTATTGTGCTGCCAAAGGTGCTGTCGTTAATTTGACCCGTGCTATGGCCTTGGATCATGGTGGCGATGGCGTCCGCATTAATTCTGTTTGCCCGAGCTTGGTCAAAACCAATATGACCAATGGTTGGCCTCAGGAAATCCGCGATAAATTCAATGAACGTATCGCCTTAGGTCGTGCTGCTGAACCTGAAGAAGTCGCTGCCGTTATGGCCTTTTTGGCTTCTGATGATGCCAGCTTTATTAATGGTGCTAATATTCCGGTTGATGGTGGCGCTACCGCCTCTGATGGC**GCT**CCTAAAATTGTTTAA

Q247A+V139Q *Smbdh*

ATGCATCACCATCATCATCATGAGAATCTGTACTTTCAGGGTCGTTTCGATAATAAAGTTGTCGTTATCACCGGTGCCGGCAATGGTATGGGCGAAGCCGCTGCCCGTCGCTTTTCTGCTGAAGGTGCCATTGTCGTTTTGGCTGATTGGGCCAAAGAAGCTGTCGATAAAGTTGCTGCCTCTTTGCCGAAAGGCCGTGCTATGGCCGTCCATATTGATGTTAGCGATCATGTCGCCGTTGAAAAAATGATGAATGAAGTTGCTGAAAAATTGGGTCGCATTGATGTCTTGTTGAATAATGCCGGTGTCCATGTTGCTGGCTCTGTTTTGGAAACCAGCATTGATGATTGGCGTCGCATTGCCGGTGTCGATATTGATGGCGTCGTCTTTTGTAGCAAATTTGCTTTGCCTCATTTGTTGAAAACCAAAGGCTGCATTGTCAATACCGCCTCT**CAA**AGCGGTTTGGGTGGCGATTGGGGCGCTGCCTATTATTGTGCTGCCAAAGGTGCTGTCGTTAATTTGACCCGTGCTATGGCCTTGGATCATGGTGGCGATGGCGTCCGCATTAATTCTGTTTGCCCGAGCTTGGTCAAAACCAATATGACCAATGGTTGGCCTCAGGAAATCCGCGATAAATTCAATGAACGTATCGCCTTAGGTCGTGCTGCTGAACCTGAAGAAGTCGCTGCCGTTATGGCCTTTTTGGCTTCTGATGATGCCAGCTTTATTAATGGTGCTAATATTCCGGTTGATGGTGGCGCTACCGCCTCTGATGGC**GCT**CCTAAAATTGTTTAA

11aa-Ins-*Smbdh*

ATGCATCACCATCATCATCATGAGAATCTGTACTTTCAGGGTCGTTTCGATAATAAAGTTGTCGTTATCACCGGTGCCGGCAATGGTATGGGCGAAGCCGCTGCCCGTCGCTTTTCTGCTGAAGGTGCCATTGTCGTTTTGGCTGATTGGGCCAAAGAAGCTGTCGATAAAGTTGCTGCCTCTTTGCCGAAAGGCCGTGCTATGGCCGTCCATATTGATGTTAGCGATCATGTCGCCGTTGAAAAAATGATGAATGAAGTTGCTGAAAAATTGGGTCGCATTGATGTCTTGTTGAATAATGCCGGTGTCCATGTTGCTGGCTCTGTTTTGGAAACCAGCATTGATGATTGGCGTCGCATTGCCGGTGTCGATATTGATGGCGTCGTCTTTTGTAGCAAATTTGCTTTGCCTCATTTGTTGAAAACCAAAGGCTGCATTGTCAATACCGCCTCTGTTAGCGGTTTGGGTGGCGATTGGGGCGCTGCCTATTATTGTGCTGCCAAAGGTGCTGTCGTTAATTTGACCCGTGCTATGGCCTTGGATCATGGTGGCGATGGCGTCCGCATTAATTCTGTTTGCCCGAGCTTGGTCAAAACCAATATGACCAATGGTTGGCCTCAGGAAATC**TCTGAAGCGGCGGGAAAACCTCTGGGTTATGGAACCGAAACA**TTCAATGAACGTATCGCCTTAGGTCGTGCTGCTGAACCTGAAGAAGTCGCTGCCGTTATGGCCTTTTTGGCTTCTGATGATGCCAGCTTTATTAATGGTGCTAATATTCCGGTTGATGGTGGCGCTACCGCCTCTGATGGCCAACCTAAAATTGTTTAA

N-term *Ecbdh*

ATGCATCACCATCATCATCATGAGAATCTGTACTTTCAGGGTCAGAAGGTGGCGCTGGTCACTGGTTCGGGTCAAGGTATAGGTAAGGCTATCGCTCTGCGGCTGGTGAAAGACGGCTTTGCCGTTGCTATTGCTGATTATAATGACGAAACCGCCCGGGCAGTTGCCGATGAGATTATTCGCAATGGCGGCAACGCGGTTGCCGTCAAAGTCGATGTTTCAGACCGCGATCAGGTCTTCGCTGCCGTTGAAAAAGCCCGCACTGCATTGGGAGGTTTTAACGTGATTGTGAATAACGCGGGTATTGCCCCTTCTACACCCATAGAATCGATTACACCGGAAATAGTCGATAAAGTTTATAATATTAATGTCAAAGGCGTTATTTGGGGTATGCAAGCCGCCATTGATGCATTTCGTAAAGAAGGCCATGGTGGAAAAATAATTAATGCTTGCAGTCAGGCTGGACATACGGGTAATCCGGAGTTGGCCGTTTATTCGAGCTCTAAATTCGCAGTGCGGGGACTGACCCAGACTGCCGCTAGAGATTTAGCTCCCCTTGGCATAACGGTTAATGCTTATTGTCCGGGGATCGTTAAAACCCCGATGTGGGCAGAAATCGATCGCCAGGTT**TCTGAAGCCGCAGGCAAACCGTTGGGTTATGGCACTGAAACC**TTTGCCAAACGTATTACGCTTGGTCGCTTGTCTGAACCCGAAGATGTGGCTGCCTGTGTTAGCTATTTGGCGGGTCCGGATTCTGATTATATGACCGGTCAGTCTCTGTTAATTGACGGTGGGATGGTATTCAATTAA

11aa-del-*Ecbdh*

ATGCATCACCATCATCATCATGAGAATCTGTACTTTCAGGGTCAGAAGGTGGCGCTGGTCACTGGTTCGGGTCAAGGTATAGGTAAGGCTATCGCTCTGCGGCTGGTGAAAGACGGCTTTGCCGTTGCTATTGCTGATTATAATGACGAAACCGCCCGGGCAGTTGCCGATGAGATTATTCGCAATGGCGGCAACGCGGTTGCCGTCAAAGTCGATGTTTCAGACCGCGATCAGGTCTTCGCTGCCGTTGAAAAAGCCCGCACTGCATTGGGAGGTTTTAACGTGATTGTGAATAACGCGGGTATTGCCCCTTCTACACCCATAGAATCGATTACACCGGAAATAGTCGATAAAGTTTATAATATTAATGTCAAAGGCGTTATTTGGGGTATGCAAGCCGCCATTGATGCATTTCGTAAAGAAGGCCATGGTGGAAAAATAATTAATGCTTGCAGTCAGGCTGGACATACGGGTAATCCGGAGTTGGCCGTTTATTCGAGCTCTAAATTCGCAGTGCGGGGACTGACCCAGACTGCCGCTAGAGATTTAGCTCCCCTTGGCATAACGGTTAATGCTTATTGTCCGGGGATCGTTAAAACCCCGATGTGGGCAGAAATCGATCGCCAGGTTCGCGATAAATTTGCCAAACGTATTACGCTTGGTCGCTTGTCTGAACCCGAAGATGTGGCTGCCTGTGTTAGCTATTTGGCGGGTCCGGATTCTGATTATATGACCGGTCAGTCTCTGTTAATTGACGGTGGGATGGTATTCAATTAA

Key:

Red text: 6X Histidine tag

Underlined text: TEV protease cleave site

**Bold underlined text:** 14aa coding α6 helix sequence

Green text: Point mutation
