# Supplementary material for: Phylogenetics-based identification and characterization of a superior 2,3-butanediol dehydrogenase for Zymomonas mobilis expression
Source: Biotechnol Biofuels. 2020 Nov 10;13:186. doi: 10.1186/s13068-020-01820-x (PMC7656694; doi:10.1186/s13068-020-01820-x)
Supplement: Supplementary file 6 — Additional file 6. 2,3-butanediol dehydrogenase gene sequences used in the study. [file 13068_2020_1820_MOESM6_ESM.docx]

**Additional file 7.** Primers used in this study.

| **Primer name** | **Primer sequence (5' - 3')** |
| --- | --- |
| SV-90 | CAG GTC ACC AGC TCA CCG |
| SV-91 | CGA AGG TGA GCC AGT GTG A |
| SV-161 | ATC ATC ATC ATG AGA ATC TGT ACT TTC AGG GTC GTT TCG ATA ATA AAG TTG |
| SV-162 | CTC ATG ATG ATG ATG GTG ATG CAT GGT ACC TGC TTA CTC CAT ATA TTC |
| SV-274 | CCG CCT CTG ATG GCG CTC CTA AAA TTG TTT AAG |
| SV-275 | GCC ATC AGA GGC GGT AGC GCC ACC ATC AAC C |
| SV-279 | TCA ATA CCG CCT CTC AAA GCG GTT TGG GTG |
| SV-280 | GAG AGG CGG TAT TGA CAA TGC AGC C |
| SV-277 | CCT CTG GGT TAT GGA ACC GAA ACA TTC AAT GAA CGT ATC GCC |
| SV-276 | TTC CAT AAC CCA GAG GTT TTC CCG CCG CTT CAG AGA TTT CCT GAG GCC AAC C |
| SV-285 | ATG CAG AAG GTG GCG CTG |
| SV-286 | TTA ATT GAA TAC CAT CCC ACC GTC |
| SV-283 | ACC TTC TGC ATT GCT TAC TCC ATA TAT TC |
| SV-284 | TAT TCA ATT AAG GAT CCG TCG ACT AC |
| SV-293 | CTC ATG ATG ATG ATG GTG ATG CAT TGC TTA CTC CAT ATA TTC |
| SV-294 | ACC ATC ATC ATC ATG AGA ATC TGT ACT TTC AGG GTC AGA AGG TGG CGC TGG TC |
| SV-302 | GTT CGC GAT AAA TTT GCC AAA CGT ATT ACG |
| SV-303 | CAA ATT TAT CGC GAA CCT GGC GAT CGA TTT C |
