# Supplementary material for: Phylogenetics-based identification and characterization of a superior 2,3-butanediol dehydrogenase for Zymomonas mobilis expression
Source: Biotechnol Biofuels. 2020 Nov 10;13:186. doi: 10.1186/s13068-020-01820-x (PMC7656694; doi:10.1186/s13068-020-01820-x)
Supplement: Supplementary file 7 — Additional file 7. Primers used in this study. [file 13068_2020_1820_MOESM7_ESM.docx]

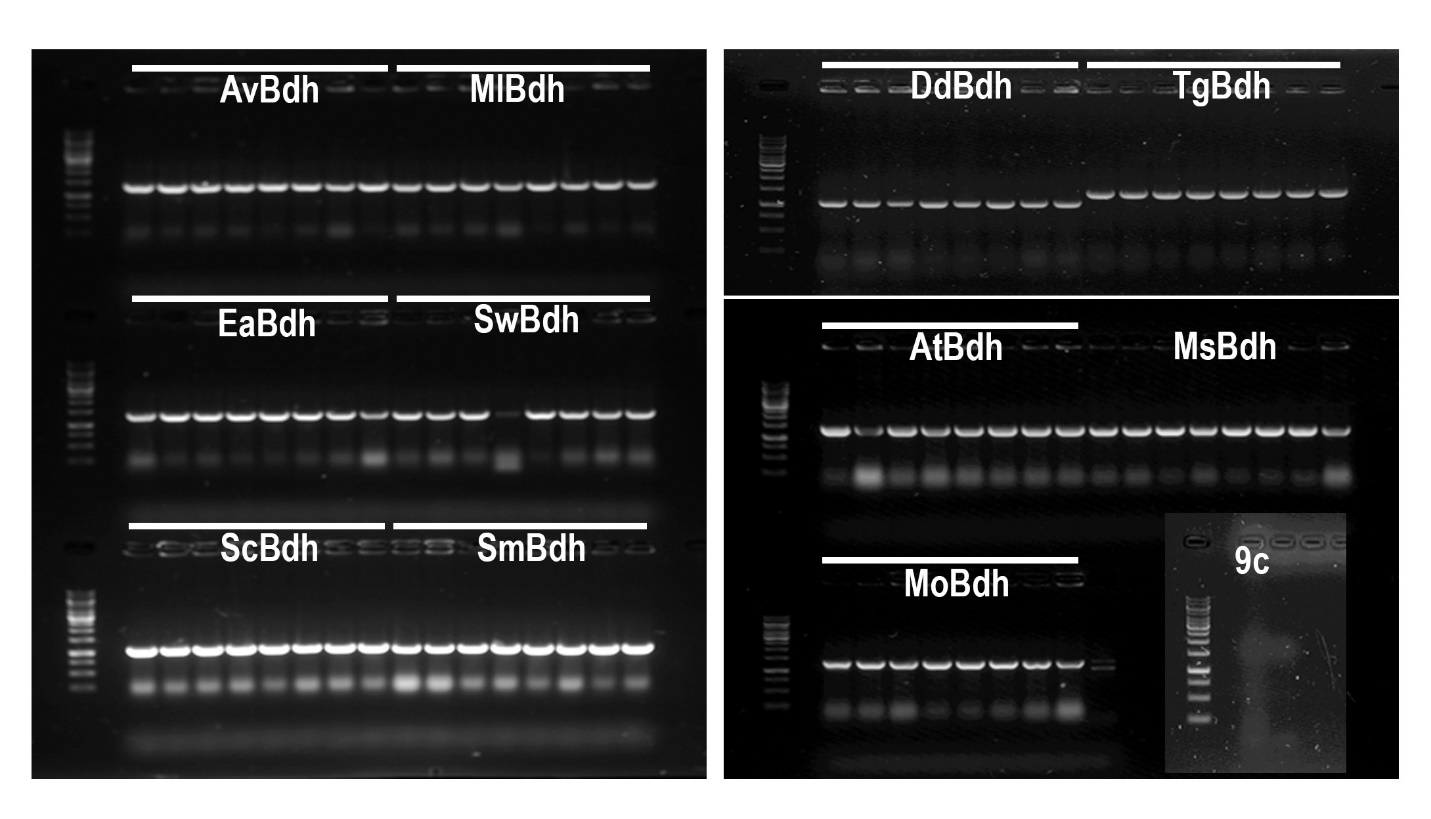


**Additional file 1.** **PCR analysis of *Z. mobilis* tranformants containing the different Bdh genes.** Eleven Bdh genes were transformed into *Z. mobilis* strain 9C and plated on RMG medium containing spectinomycin. Eight independent colonies were selected for colony PCR analysis using gene specific primers. Five microliters of the PCR products were run on 1% agarose gel and visualized using FluoChem Gel analyzer. White bar represents sets of 8 lanes for each Bdh gene. Av, *Azotobacter vinelandii*; Ml, *Micrococcus luteus;* Ea, *Erwinia amylovora;* Sw, *Staphylococcus warneri*; Sc, *Streptomyces coelicolor*; Sm, *Serratia marcescens*; Dd, *Dickeya dadantii*; At, Tg, *Thermococcus gammatolerans*; *Agrobacterium tumefaciens*; Ms, *Mycobacterium Smegmatis*; Mo, *Myroides odoratimimus*; 9c, *Z. mobilis* control strain.
